# Involvement of the VEGF signaling pathway in immunosuppression and hypoxia stress: analysis of mRNA expression in lymphocytes mediating panting in Jersey cattle under heat stress

**DOI:** 10.1186/s12917-021-02912-y

**Published:** 2021-06-07

**Authors:** Jian Wang, Yang Xiang, Shisong Jiang, Hongchang Li, Flurin Caviezel, Suporn Katawatin, Monchai Duangjinda

**Affiliations:** 1 Faculty of Veterinary Medicine, Southwest University, Chongqing, 400700 China; 2 Department of Oncology, Oxford University, Oxford, OX3 7DQ UK; 3 Department of Animal Science, Khon Kaen University, Kaen, 40002 Thailand

**Keywords:** Vascular endothelial growth factor, Immune, Lymphocytes, Dairy cattle, Heat stress

## Abstract

**Background:**

Extreme panting under heat stress threatens dairy cattle milk production. Previous research has revealed that the gas exchange-mediated respiratory drive in critically ill dairy cattle with low O_2_ saturation induces panting. Vascular endothelial growth factor (VEGF) signaling may play important roles in immunosuppression and oxidative stress during severe respiratory stress responses in heat-stressed cattle. The objectives of this study were to transcriptomically analyze mRNA expression mediating heat-induced respiratory stress-associated panting, evaluate gas exchange, screen hub genes, and verify the expression of proteins encoded by differentially expressed genes in lymphocyte pathways.

**Results:**

Jersey cattle were naturally heat-exposed. Physiological data were collected for response evaluation, and blood was collected for gas exchange and gene expression assays at 06:00, 10:00 and 14:00 continuously for 1 week. Lymphocytes were isolated from whole-blood samples for mRNA-seq and expression analysis of key pathway genes/proteins. The cattle respiration rates differed with time, averaging 51 bpm at 06:00, 76 bpm at 10:00, and 121 bpm at 14:00 (*p* < 0.05). Gas exchange analysis showed that both pH and pCO_2_ differed with time: they were 7.41 and 41 mmHg at 06:00, 7.45 and 37.5 mmHg at 10:00, and 7.49 and 33 mmHg at 14:00, respectively (*p* < 0.01). Sixteen heat-related differentially expressed genes (DEGs; 13 upregulated and 3 downregulated) were screened between 212 DEGs and 1370 heat stress-affected genes. Kyoto Encyclopedia of Genes and Genomes (KEGG) hub gene functional analysis annotated eleven genes to signal transduction, six genes to the immune response, and five genes to the endocrine response, including both prostaglandin-endoperoxide synthase 2 (PTGS2) and VEGF. Gene Ontology (GO) functional enrichment analysis revealed that oxygen regulation was associated with the phosphorus metabolic process, response to oxygen levels, response to decreased oxygen levels, response to hypoxia and cytokine activity terms. The main signaling pathways were the VEGF, hypoxia inducible factor-1(HIF-1), cytokine-cytokine receptor interaction and TNF pathways. Four genes involved Integrin beta 3 (ITBG3), PTGS2, VEGF, and myosin light chain 9 (MYL9) among the 16 genes related to immunosuppression, oxidative stress, and endocrine dysfunction were identified as participants in the VEGF signaling pathway and oxygenation.

**Conclusion:**

These findings help elucidate the underlying immune and oxygen regulation mechanisms associated with the VEGF signaling pathway in heat-stressed dairy cattle.

**Supplementary Information:**

The online version contains supplementary material available at 10.1186/s12917-021-02912-y.

## Background

The specific physiological responses of homeothermal animals under heat stress have been found to involve hypoxia, oxidative stress, decreased immunity, respiratory acidosis, and electrolyte disturbances. A previous study has shown that the physiological panting response of dairy cattle is affected by interactions between breed and the environment [1, 2]. Panting related to the chemical and metabolic acid-base balance of animals that occurs under a range of environmental stressors affecting respiratory condition is based on the mechanism of H^+^ equivalent exchange. For instance, panting reduces systemic arterial pO_2_ and Hb O_2_ (HbO_2_) saturation but increases the partial pressure of CO_2_, leading to increased H^+^ accumulation [3]. Furthermore, under heat stress, dairy cattle attempt to dissipate heat by panting. The mechanism of oxygenation is insufficient for immune cell metabolism, although quick breathing increases gas exchange between the environment and lungs. In addition, a previous transcriptomic analysis has revealed that 2'-5'-oligoadenylate synthetase 2 (OAS2), MX dynamin like GTPase 2 (MX2), interferon induced protein with tetratricopeptide repeats 5 (IFIT5) and TGFB2 are potential regulatory genes related to heat tolerance in Holstein dairy cattle [4]. However, transcriptomic analysis of messenger RNA (mRNA) sequences with a focus on the immune response and oxidative stress in dairy cattle under heat stress has not been performed.

Immune responses assist dairy cattle in maintaining a healthy microenvironment by releasing cytokines and activating genes to protect tissue and cells from damage caused by heat stress [5]. In line with the dynamic nature of heat stress, two phases are recognized: an early inflammatory phase and a late immunosuppressive phase [6]. Shalova et al. [7] found that the early phase is characterized by leukocyte activation, a cytokine storm, and a systemic inflammatory response, while the late phase is characterized by immunosuppression involving leukocyte deactivation, increased risk of secondary infection, and high mortality during heat stress. For example, it would effected in the early inflammation on immune function due to increased white blood cell counts when dairy cattle are under heat stress [8]. Furthermore, previous studies have reported that the effects of hypoxia on cytokine gene expression, inflammation and immunosuppression involve VEGF [9], PTGS2 [9, 10], endothelin-1 (EDN1) [11], nuclear receptor subfamily 4 group A member 2 (NR4A2) [12–14], C-X-C motif chemokine ligand 8 (CXCL8) [15, 16], interleukin 1 alpha (IL1A) [17] and MYL9 [18]. It is possible for heat to induce both hypoxia and immune responses to affect the expression of common genes with related functions.

In addition, oxidative stress, a physiological response to heat stress, is provoked in dairy cattle by oxygen metabolism-related enzymes [19]. Several studies have concluded that exposure of dairy cattle to heat stress induces oxidative stress, which can lead to cytotoxicity [20]. It is well known that biological macromolecules can be damaged by oxidative stress, which interferes with metabolic and physiological pathways [21]. Moreover, the symptoms of heat stress have been suggested to be similar to those of oxidative stress because of correspondences in the expressed genes, including genes encoding heat-shock proteins and antioxidant enzymes [22, 23]. It is possible that the VEGF signaling pathway plays an important role in regulating oxygen levels.

Therefore, it is interesting to explore whether the effects of immune-mediated destruction of cells under heat stress on gene expression help to maintain homeostasis in dairy cattle. Thus far, the gene regulation mechanism of heat-induced panting with regard to the mRNA expression levels of hub genes that regulate immunosuppression and oxygen stress in blood immune cells in dairy cattle under heat stress has remained unknown. It has been hypothesized that the heat stress-induced panting response of dairy cattle may be regulated by hub genes within the VEGF signaling pathway that play important roles in immunosuppression, oxidative stress, and the endocrine system.

## Results

### Meteorological data, physiological responses, and gas exchange

Meteorological data, namely, the dry-bulb temperature, wet-bulb temperature, and relative humidity, significantly differed with the temperature humidity index (THI) (*p* < 0.01) (Table 1). All physiological responses, including respiratory rate (RR), rectal temperature (RT), and skin temperature (ST), increased as the THI increased; the strongest response appeared at 14:00 when THI was highest (THI = 88) (*p* < 0.01) (Table 1). Significant differences in were observed; the RR was 51 bpm at 06:00(THI77), 76 bpm at 10:00(THI82) and 121 bpm at 14:00(THI88) (*p* < 0.01). The gas exchange data showed that both pH and pCO_2_ significantly differed and were affected by the THI (*p* < 0.01). The pH values increased as the THI increased, while the pCO_2_ values decreased (Table 1).

**Table 1:** Meteorological data, physiological responses and gas exchange of Jersey dairy cattle according to THI (time)

Variable	THI (time)			SEM	*P*-value
	77 (06:00)	82 (10:00)	88 (14:00)		
Indoor temperature (°C)	26.7^c^	30.2^b^	36.0^a^	0.13	< 0.01
Outdoor temperature (°C)	25.4^c^	29.6^b^	36.0^a^	0.11	< 0.01
Humidity	68.2^a^	52.0^b^	31.8^c^	0.21	< 0.01
Dry-bulb temperature (°C)	26.1^d^	29.5^c^	35.3^a^	0.12	< 0.01
Wet-bulb temperature (°C)	25.2^c^	26.5^b^	28.7^a^	0.11	< 0.01
THI	77^c^	82^b^	88^a^	0.13	< 0.01
RR (bpm)	51^c^	76 ^b^	121^a^	0.46	< 0.01
RT (°C)	38.5^c^	38.7^b^	39.6^a^	0.05	< 0.01
ST (with hair) (°C)	36.0^c^	38.2^b^	39.3^a^	0.1	< 0.01
pH	7.41^c^	7.45^b^	7.49^a^	0.05	< 0.01
pCO_2_ (mmHg)	41^a^	37.5^b^	33^c^	0.2	< 0.01

*THI* temperature humidity index, *SEM* standard error of the mean

^a, b, c,^ Means within a row with different superscripts differ (*p* < 0.01)

### RNA-seq results

The transcriptome sequencing results are shown in Table 2. From the samples in the THI = 77 (THI77) group, 61,562,712 (THI = 77_1), 59,409,952 (THI = 77_2), 67,050,696 (THI = 77_3), 58,325,238 (THI = 77_4) and 69,485,932 (THI = 77_5) raw reads were obtained. From the samples in the THI = 82 (THI82) group, 50,837,238 (THI = 82_1), 58,619,002 (THI = 82_2), 50,511,366 (THI = 82_3), 59,646,870 (THI = 82_4), and 63,367,910 (THI = 82_5) raw reads were obtained. From the samples in the THI = 88 (THI88) group, 62,085,400 (THI = 88_1), 59,070,634 (THI = 88_2), 59,661,384 (THI = 88_3), 69,221,370 (THI = 88_4), and 61,949,988 (THI = 88_5) raw reads were obtained. Clean reads were obtained by removing reads containing adapters and undetermined bases and low-quality reads from the group of raw reads. From the samples in the THI77 group, 61,069,186 (THI = 77_1), 58,867,260 (THI = 77_2), 66,516,354 (THI = 77_3), and 57,799,350 (THI = 77_4) clean reads were obtained. From the samples in the THI82 group, 50,474,676 (THI = 82_1), 58,178,126 (THI = 82_2), 50,049,474 (THI = 82_3), 59,215,152 (THI = 82_4), and 62,924,034 (THI = 82_5) clean reads were obtained. From the samples in the THI88 group, 61,551,498 (THI = 88_1), 58,633,654 (THI = 88_2), 59,176,084 (THI = 88_3), 68,728,564 (THI = 88_4), and 61,437,404 (THI = 88_5) clean reads were obtained. All obtained Q20 and Q30 values were greater than 95%, and the error rates were less than 0.05% in the THI77, THI82 and THI88 groups.

**Table 2:** Statistics from mRNA-seq for peripheral lymphocytes in Jersey cattle under heat stress

Sample	Raw reads	Raw bases	Clean reads	Clean bases	Error rate (%)	Q20 (%)	Q30 (%)	GC (%)	Total reads	Total mapped reads	Reads mapped to multiple loci	Uniquely mapped reads
THI = 77_1	61,562,712	9,276,910,694	61,069,186	9.1 Gb	0.0231	98.86	96.05	51.33	61,069,186	58,163,771 (95.24%)	1,580,043 (2.59%)	56,583,728 (92.66%)
THI = 77_2	59,409,952	8,952,561,296	58,867,260	8.8 Gb	0.0230	98.90	96.19	51.49	58,867,260	56,022,325 (95.17%)	1,623,579 (2.76%)	54,398,746 (92.41%)
THI = 77_3	67,050,696	10,103,532,968	66,516,354	9.9 Gb	0.0228	98.97	96.41	51.37	66,516,354	63,369,583 (95.27%)	1,426,145 (2.14%)	61,943,438 (93.13%)
THI = 77_4	58,325,238	8,788,851,462	57,799,350	8.6 Gb	0.0228	98.95	96.37	51.41	57,799,350	55,156,163 (95.43%)	1,315,965 (2.28%)	53,840,198 (93.15%)
THI = 77_5	69,485,932	8,957,561,292	55,867,460	8.8 Gb	0.0229	98.94	96.29	51.48	57,867,265	56,214,338 (95.17%)	1,633,578 (2.76%)	53,396,721 (92.41%)
THI = 82_1	50,837,238	7,660,716,518	50,474,676	7.5 Gb	0.0229	98.91	96.22	51.25	50,474,676	48,100,973 (95.3%)	1,022,965 (2.03%)	47,078,008 (93.27%)
THI = 82_2	58,619,002	8,833,461,136	58,178,126	8.7 Gb	0.0228	98.96	96.38	51.84	58,178,126	55,529,590 (95.45%)	1,621,922 (2.79%)	53,907,668 (92.66%)
THI = 82_3	50,511,366	7,611,557,460	50,049,474	7.4 Gb	0.0232	98.79	95.90	51.77	50,049,474	47,388,832 (94.68%)	1,201,415 (2.4%)	46,187,417 (92.28%)
THI = 82_4	59,646,870	8,988,057,544	59,215,152	8.8 Gb	0.0227	98.99	96.45	51.40	59,215,152	56,428,062 (95.29%)	1,484,362 (2.51%)	54,943,700 (92.79%)
THI = 82_5	63,367,910	9,548,852,684	62,924,034	9.4 Gb	0.0228	98.98	96.44	52.07	62,924,034	59,770,598 (94.99%)	1,575,192 (2.5%)	58,195,406 (92.49%)
THI = 88_1	62,085,400	9,355,746,764	61,551,498	9.2 Gb	0.0229	98.91	96.25	52.02	61,551,498	58,767,150 (95.48%)	1,654,125 (2.69%)	57,113,025 (92.79%)
THI = 88_2	59,070,634	8,901,656,122	58,633,654	8.7 Gb	0.0227	99.00	96.50	52.42	58,633,654	55,752,783 (95.09%)	1,825,538 (3.11%)	53,927,245 (91.97%)
THI = 88_3	59,661,384	8,990,075,482	59,176,084	8.8 Gb	0.0229	98.93	96.30	51.77	59,176,084	56,335,828 (95.2%)	1,601,284 (2.71%)	54,734,544 (92.49%)
THI = 88_4	69,221,370	10,430,405,822	68,728,564	10.2 Gb	0.0227	98.99	96.45	50.44	68,728,564	65,469,192 (95.26%)	1,737,699 (2.53%)	63,731,493 (92.73%)
THI = 88_5	61,949,988	9,335,032,514	61,437,404	9.2 Gb	0.0230	98.90	96.19	51.46	61,437,404	58,563,815 (95.32%)	1,402,307 (2.28%)	57,161,508 (93.04%)

### DEG analysis and screening of 16 heat-related DEGs in lymphocytes of Jersey dairy cattle affected by the THI under heat stress

**Fig. 1:**
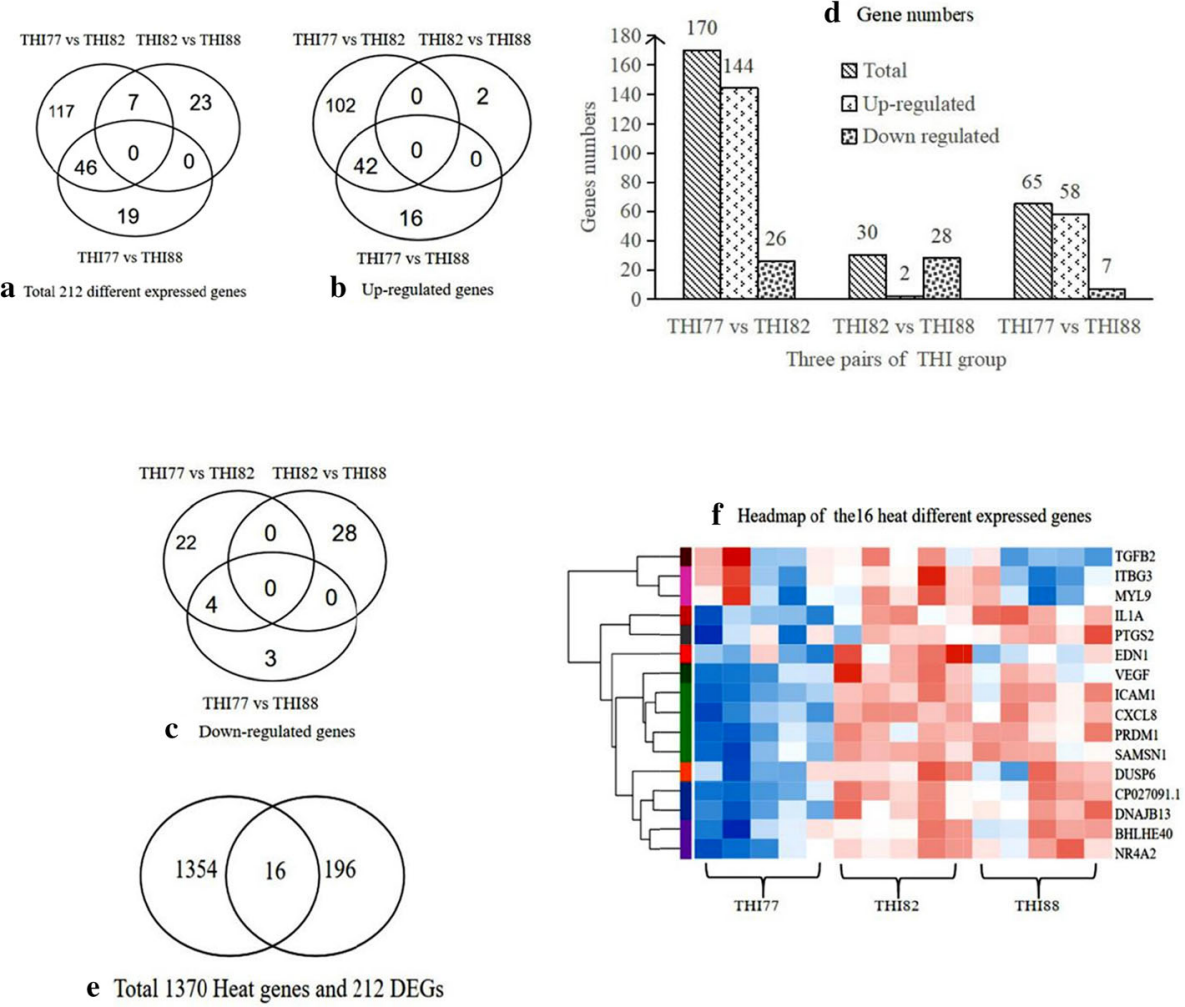
DEGs were subjected to Venn analysis using normalized read counts from three pairs of groups: THI77 vs THI82, THI82 vs THI88 and THI77 vs THI88. The 16 heat-related DEGs were screened, and a heatmap was created. **a**. Venn statistics showing the total DEGs in each pair of THI groups. **b**. Venn statistics showing the upregulated DEGs in each pair of THI groups. **c**. Venn statistics showing the downregulated DEGs in each pair of THI groups. **d**. Diagram showing the total numbers of DEGs and the numbers of upregulated and downregulated DEGs in each pair of THI groups. **e**. Venn diagram showing the 16 heat-related DEGs screened between 212 DEGs and 1370 heat-related genes. **f**. Heatmap of the 16heat-related DEGs

The DEGs between each pair of groups were revealed via differential expression analysis using normalized read counts and are shown in Fig. 1A. The upregulated DEGs between each pair of groups are shown in Fig. 1B, while the downregulated DEGs are shown in Fig. 1C. RNA-seq analysis screened 170 DEGs in the THI82 group compared to the control group of THI77 cells, of which 26 genes were downregulated and 144 were upregulated. Additionally, RNA-seq analysis identified 30 DEGs in the THI88 group compared to the control group of THI82 cells, of which 2 were upregulated and 28 were downregulated. Finally, RNA-seq analysis identified 65 DEGs in the THI77 group compared to the control group of THI88 cells, of which 58 were upregulated and 5 were downregulated (Fig. 1D). Subsequently, 16 heat-related DEGs were screened by Venn analysis between 1370 heat-related genes and 212 DEGs (Fig. 1E). Furthermore, 16 heat-related DEGs (including 13 upregulated DEGs and 3 downregulated DEGs) were obtained using the DESeq2 package (version 1.10.1) according to the values of a |log2 fold change| ≥ 1 and a *p* < 0.05 (Fig. 1F, Table 3).

**Table 3:** The gene description, KEGG Orthology (KO)ID, KO name for the 16 heat-related DEGs

Gene ID	Gene name	Gene description	KEGG Orthology (KO) ID	KO name	P-value	Adjusted p-value	Regulation direction
ENSBTAG00000005359	TGFB2	Transforming growth factor beta 2 [Source:HGNC Symbol;Acc:HGNC:11768]	–	–	3.55E-06	0.00228	down
ENSBTAG00000009987	ITBG3	Alpha IIb Beta 3 Integrin [platelet alpha granule membrane]	–	–	6.04E-06	0.00310	down
ENSBTAG00000011473	MYL9	Myosin light chain 9 [Source:HGNC Symbol;Acc:HGNC:15754]	K12755	MYL9	6.22E-04	0.03617	up
ENSBTAG00000010349	IL1A	Interleukin 1 alpha	K04383	IL1A	1.48E-05	0.00470	down
ENSBTAG00000014127	PTGS2	Prostaglandin-endoperoxide synthase 2 [Source:HGNC Symbol;Acc:HGNC:9605]	K11987	PTGS2, COX2	4.81E-08	0.00003	up
ENSBTAG00000008096	EDN1	Endothelin 1 [Source:HGNC Symbol;Acc:HGNC:3176]	K16366	EDN1	9.38E-07	0.00030	up
ENSBTAG00000005339	VEGF	Vascular endothelial growth factor A [Source:VGNC Symbol;Acc:VGNC:55927	K05448	VEGFA	1.99E-08	0.00001	up
ENSBTAG00000010303	ICAMI	–	K06490	ICAM1, CD54	3.81E-08	0.00001	up
ENSBTAG00000019716	CXCL8	Interleukin-8 [Source:UniProtKB/Swiss-Prot;Acc:P79255]	K10030	IL8, CXCL8	IL-8	0.00829	up
ENSBTAG00000000816	PRDM1	PR/SET domain 1 [Source:HGNC Symbol;Acc:HGNC:9346]	–	–	8.46E-06	0.00163	up
ENSBTAG00000002623	SAMSN1	SAM domain, SH3 domain and nuclear localization signals 1 [Source:HGNC Symbol;Acc:HGNC:10528]	–	–	1.95E-07	0.00006	up
ENSBTAG00000004587	DUSP6	Dual-specificity phosphatase 6 [Source:HGNC Symbol;Acc:HGNC:3072]	K04459	DUSP, MKP	6.09E-06	0.00091	up
ENSBTAG00000047561	CP027091.1	–	K05448	VEGFA	5.01E-05	0.00629	up
ENSBTAG00000003682	DNAJB13	DnaJ heat shock protein family (Hsp40) member B13	K09519	DNAJB13	6.74E-07	0.00024	up
ENSBTAG00000009863	BHLHE40	Basic helix-loop-helix family member e40 [Source:HGNC Symbol;Acc:HGNC:1046]	K03729	BHLHB2, DEC1	2.41E-08	0.00001	up
ENSBTAG00000003650	NR4A2	Nuclear receptor subfamily 4 group A member 2 [Source:HGNC Symbol;Acc:HGNC:7981]	K08558	NR4A2, NURR1	6.81E-16	0.00000	up

### Functional annotation and enrichment of the 16 heat-related DEGs by KEGG analysis

**Fig. 2:**
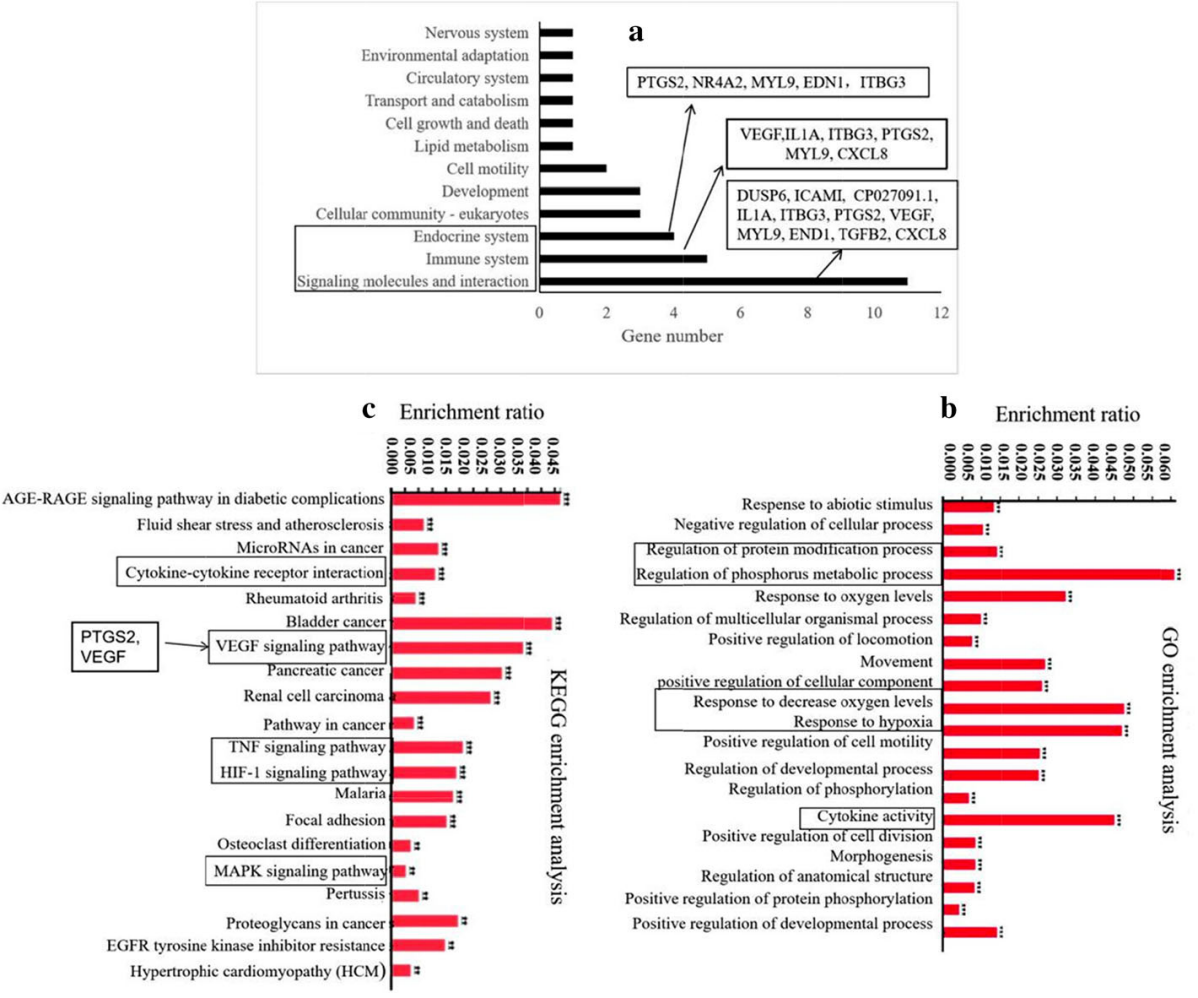
GO and KEGG functional enrichment of 16 heat-related DEGs. **a** The main annotated functions were associated with the endocrine system (PTGS2, NR4A2, MYL9, EDN1and ITGB3), the immunesystem (VEGF, IL1A, ITGB3, PTGS2, MYL9 and CXCL8) and signaling molecules and interaction(11 genes). **b** The enriched GO terms included the regulation of phosphorus metabolic process, response to oxygen levels, response to decreased oxygen levels, response to hypoxia and cytokine activity terms. **c** The enriched KEGG pathways included the cytokine-cytokine receptor interaction pathway, the VEGF signaling pathway, the TNF signaling pathway and the HIF-1 signaling pathway

Functional annotation and enrichment of the 16 heat-related DEGs revealed that immunosuppression, oxidative stress, and endocrine disorder are effected by heat stress. The KEGG functional annotations focused mainly on three major functions: signal transduction for eleven genes (AF356445.1(DUP6), intercellular adhesion molecule 1(ICAM1), CP027091.1, IL1A, ITGB3, PTGS2, VEGF, MYL9, HNHc-like endonuclease (END1), TGFB2, and CXCL8), the immune system for six genes (VEGF, IL1A, ITGB3, PTGS2, MYL9, and CXCL8) and the endocrine system for five genes (PTGS2, NR4A2, MYL9, EDN1, and ITBG3) (Fig. 2A). Some of the 16 heat-related DEGs were enriched in the following KEGG pathways: the AGE-RAGE signaling pathway in diabetic complications (enriched genes: PTGS2, VEGF, IL1A, EDN1, TGFB2, CXCL8, and ICAM1), the VEGF signaling pathway (map04370) (enriched genes: vascular endothelial growth factor A [VEGFA], PTGS2 and CP027091.1), cytokine-cytokine receptor interactions (enriched genes: IL1A, VEGFA, CXCL8 and CP027091.1), the TNF signaling pathway (map04668) (enriched genes: EDN1, ICAM1 and PTGS2), the hypoxia inducible factor-1 (HIF-1) signaling pathway (map04066) (enriched genes: VEGFA, EDN1 and CP027091.1), the MAPK signaling pathway (map04010) (enriched genes: IL1A and DUSP6), and EGFR tyrosine kinase inhibitor resistance (map01521) (enriched gene: CP027091.1) (Fig. 2C).

### Functional annotation and enrichment of the 16 heat-related DEGs by GO analysis

The top five enriched process terms in GO analysis were the phosphorus metabolic process term, the response to oxygen levels term, the response to decreased oxygen levels term, the response to hypoxia term and the cytokine activation term (Fig. 2B). The genes enriched for phosphorus metabolic process-related GO functions included SAM domain, SH3 domain and nuclear localization signals 1(SAMSN1)(GO:0050732), DUSP6(GO:0004725) and CP027091.1 (GO:0050731); those enriched for oxygen balance-related GO functions included VEGFA (GO:0043117), EDN1 (GO:0007589 and GO:0019229), NR4A2 (GO:0034599 and GO:0043576), and PTGS2 (GO:0006954, GO:0071347 and GO:0006979); those enriched for hypoxia-related GO functions included VEGFA (GO:0071456), EDN1 (GO:0001666), NR4A2 (GO:0001666) and CP027091.1 (GO:0071456); and those enriched for cytokine activation-related GO terms included IL1A (GO:0005125), VEGFA (GO:0005125), EDN1 (GO:0005125), CXCL8 (GO:0008009), and CP027091.1 (GO:0005125) (Addtional 1).

### Q-PCR validation

**Fig. 3:**
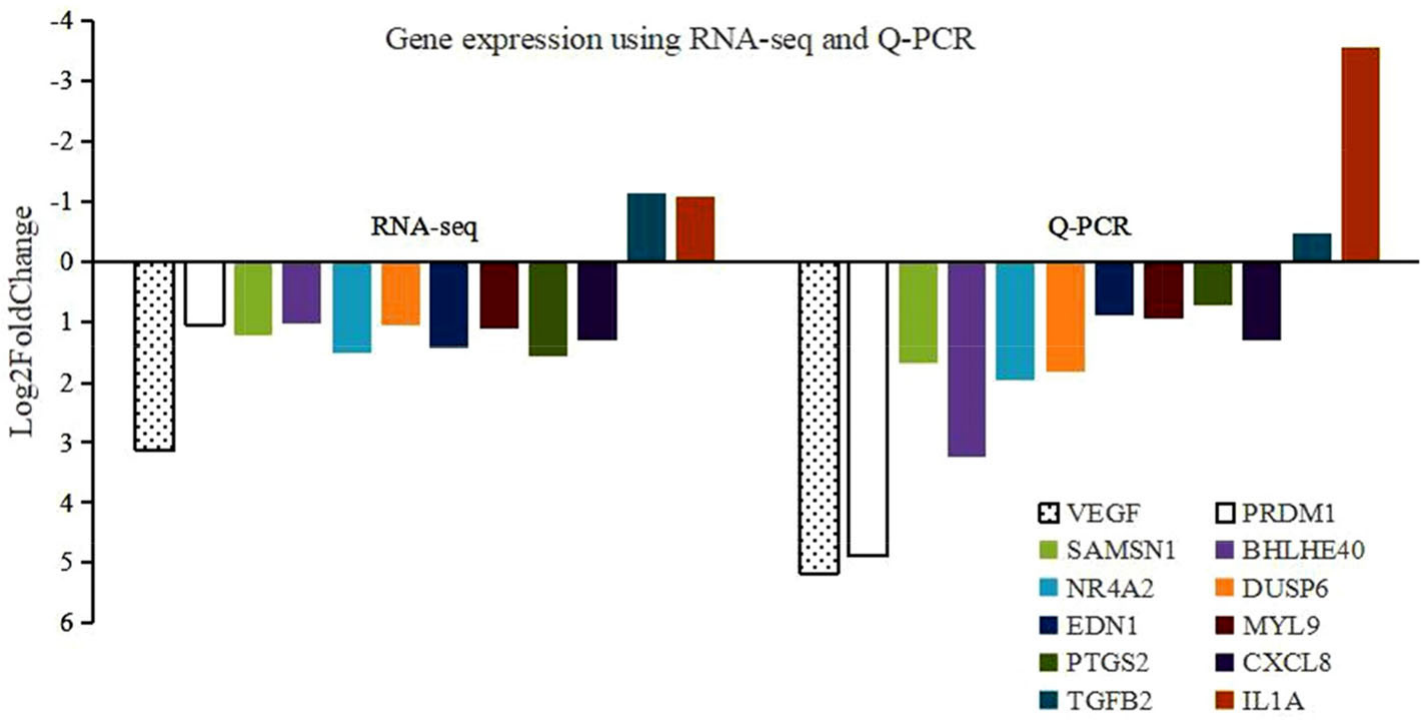
Heat-related DEG expression determined by RNA-seq and Q-PCR

To verify the reliability of the transcriptome sequencing results in this study, Q-PCR was used to detect the expression of heat-related DEGs. Compared to the control group, the experimental group exhibited increased mRNA expression of VEGF,  PR/SET domain 1 (PRDM1), SAMSN1, basic helix-loop-helix family member e40 (BHLHE40), NR4A2, DUSP6, EDN1, MYL9, prostaglandin-endoperoxide synthase 2 (PTGS2), CXCL8 and IL1A. These results were consistent with the RNA-seq results (Fig. 3).

### Strong heat stress-mediated activation of heat-related DEG coexpression and upregulation of the gene expression of VEGF and PTGS2 in the VEGF pathway in blood-derived lymphocytes

**Fig. 4:**
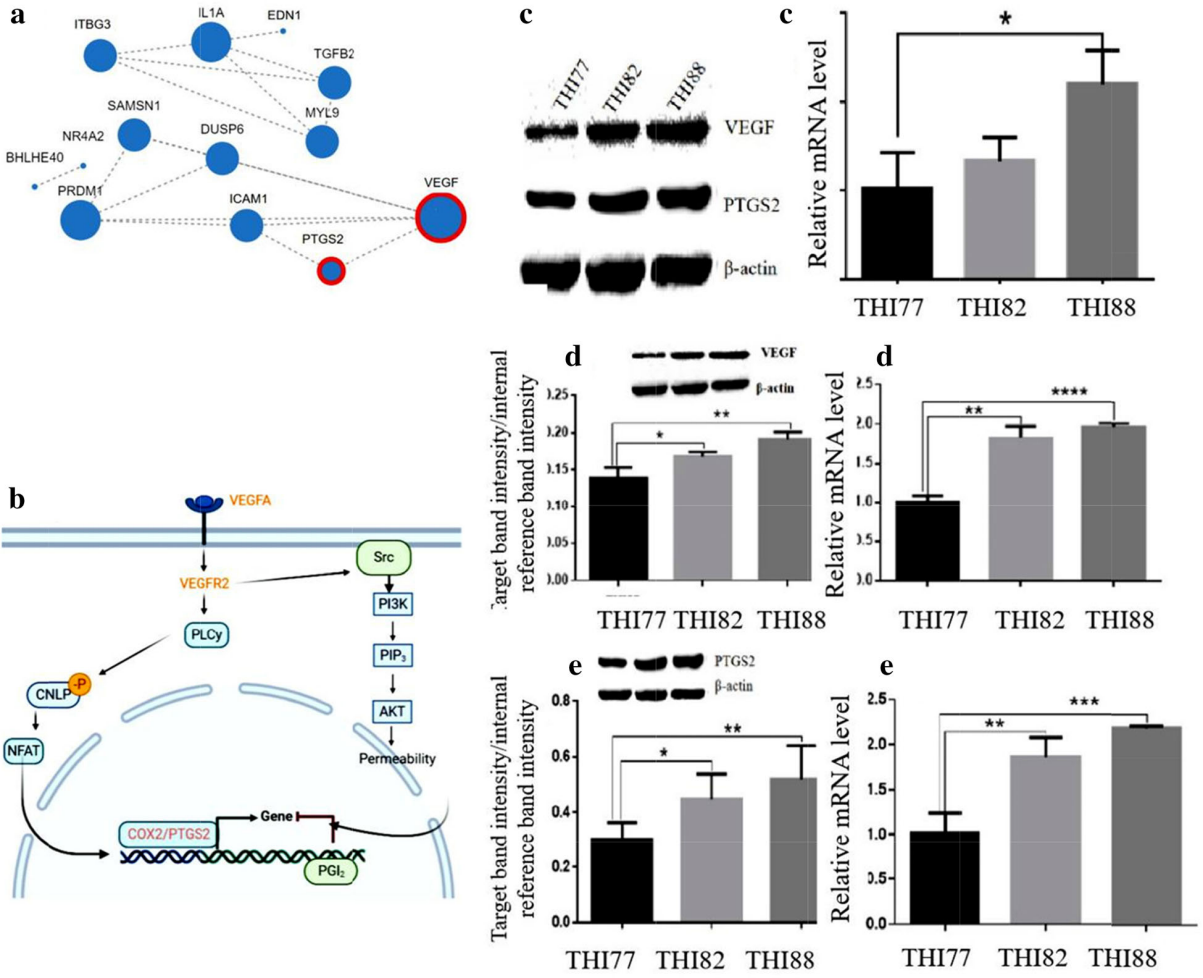
Heat-related DEG coexpression network and the expression of VEGF and PTGS2 in the VEGF pathway. **a** Coexpression network diagram of the 16 heat-related DEGs, with a positive relationship between PTGS2 and VEGF. **b** Diagram of the VEGF signaling pathway showing that VEGFA binds VEGFR2 at the membrane before triggering PLCy-CNLP-NFAT signaling to activate expression of the target gene COX2/PTGS2. **c** Verification of VEGF and PTGS2 expression. Scanned images of the protein levels of both VEGF and PTGS2 under different THIs (THI77, THI82, and THI88). c) Changes in relative mRNA expression determined using Q-PCR. **d** Changes in VEGF expression in lymphocte caused by different THIs (THI77, THI82, and THI88). d) Changes in relative mRNA expression determined using Q-PCR. **e** Changes in PTGS2 expression in lymphocytes caused by different THIs (THI77, THI82, and THI88). e) Changes in relative mRNA expression determined using Q-PCR

A coexpression network was constructed for the 16 heat-related DEGs in the blood-derived lymphocytes of Jersey dairy cattle affected by heat stress. This network included TGFB2, ITBG3, VEGF, PTGS2, NR4A2, EDN1, DUSP6, IL1A, MYL9, PRDM1, SAMSN1, BHLHE40 and ICAM1. The results showed that the VEGF gene was strongly related to other genes in the VEGF signaling pathway, including the PTGS2 gene (Fig. 4A). The results also showed that the VEGF signaling pathway was activated in the blood-derived lymphocytes of Jersey dairy cattle (*Bos taurus*). VEGFR-2 is the major mediator of VEGF (VEGFA)-driven responses in lymphocytes. Binding of VEGFA to VEGFR-2 leads to dimerization of the receptor, triggering intracellular activation of the PLCy and IP3-Ca + kinase- cyclooxygenase 2 (COX2) pathways of arachidonic acid metabolism. Subsequently, the initiation of COX2/PTGS2 gene expression increases permeability (Fig. 4B). The levels of both the VEGF and PTGS2 proteins are shown (Fig. 4C). The relative mRNA expression levels determined using Q-PCR were significantly different (*p* < 0.001) (Fig. 4c). Furthermore, both the gene and protein expression of VEGF and PTGS2 increased significantly in association with high values of THI (*p* < 0.001) (Fig. 4D, d, E, e).

## Discussion

The effects of heat stress on the heat-tolerant Jersey cattle breed (*Bos taurus*) have been investigated by Smith et al. [24]. In this study, an animal model of heat stress was successfully induced by an average temperature of 30.3 °C and an average humidity of 68% from July to August in Chongqing (29.4316°N, 106.9123°E). It was confirmed that the Jersey dairy cattle initiated panting for evaporative heat dissipation. The RR was significantly affected by THI: it was 51 bpm at THI77, 76 bpm at THI82, and 121 bpm at THI88. In addition, the gas exchange parameters of pH and pCO_2_ in the blood of Jersey dairy cattle were significantly affected: they were 7.41 and 41 mmHg at THI77, 7.45 and 37.5 mmHg at THI82, and 7.49 and 33 mmHg at THI88, respectively. Furthermore, the findings were consistent with the following standard categories: THI < 72, no stress; 72 < THI < 79, vasodilation and increased RR; and 80 < THI < 90, increased RR or even panting [2].

In this study, transcriptome sequencing analysis screened 212 DEGs and 1370 heat-related genes in the lymphocytes of Jersey dairy cattle naturally exposed to hot environmental conditions of THI77, THI82 and THI88. Ultimately, 16 heat-related DEGs were screened from the 212 DEGs and 1370 heat-related genes, including transforming growth factor beta 2 (TGFB2), ITBG3, VEGF, CP027091.1, PTGS2, NR4A2, EDN1, DUSP6, IL1A, CXCL8, ICAM1, MYL9, DnaJ heat shock protein family (Hsp40) member B13(DNAJB13), PRDM1, SAMSN1 and BHLHE40. The functional annotation results revealed that the 16 heat-related DEGs were associated with the response to hypoxia, oxidative stress, the immune response, inflammation and endocrine disorder. Likewise, six genes were annotated to the immune system, including VEGF, IL1A, ITBG3, PTGS2, MYL9 and CXCL8; five genes were annotated to the endocrine system, including PTGS2, NR4A2, MYL9, EDN1, and ITBG3; and eleven genes were annotated to signal transduction, including DUP6, ICAM1, CP027091.1, IL1A, ITBG3, PTGS2, VEGF, MYL9, END1, TGFB2 and CXCL8.

In a previous study, hypoxia was found to affect cytokine gene expression, inflammation and immunosuppression in immune cells in a manner that involved VEGF, PTGS2, EDN1, CXCL8, IL1A and MYL9. Hypoxia has also been found to affect the expression of the gene encoding EDN1 in U87 glioma cells [11] and the genes encoding PTGS2 and VEGFA in breast cancer and melanoma cells [9]. Overproduction of PTGS2 has also been observed in lung cancer for immunosuppression [10]. Furthermore, HIF-1 is activated in response to hypoxia to modulate the expression of genes such as VEGF and COX-2 via cytokines and chemokines. For example, endothelin-2 (EDN2) has been investigated in macrophages and cancer cells [9]. An investigation has also proven that MYL9 is a ligand activated by CD69 on leukocytes that is strongly detected inside blood vessels in inflamed lungs [18]. In addition, the IL1A gene plays a role in vascular regulation and hematopoiesis induced by hypoxia, but factor 1α (HIF-1α) induces the cytokine CXCL8, which activates heat shock transcription via AKT/mTOR/STAT3 pathways [15–17]. Moreover, ITGB3 (α_V_β_3_), a type of integrin that consists of two components, integrin alpha V and integrin beta 3 (CD61), is a receptor for phagocytosis on macrophages. ITBG3 is physically and functionally associated with important therapeutic targets [25]. The findings in this study show that ITBG3, PTGS2, VEGF and MYL9 are hub genes that are possible biological markers of the immune response in dairy cattle under heat stress.

Oxidative stress is provoked in dairy cattle as a physiological response to heat stress [19]. Several studies have investigated whether exposure of dairy cattle to heat stress can lead to enhanced ROS production, oxidative stress and cytotoxicity [20]. Oxidative stress can interfere with metabolic and physiological pathways by damaging biological macromolecules [21]. Moreover, heat stress and oxidative stress induce very similar gene expression patterns in dairy cattle; for example, they induce heat-shock proteins and antioxidant enzymes [22]. The results of this study show that the gene expression of VEGF and PTGS2 in lymphocytes significantly increases in order to defend against oxidative stress in Jersey dairy cattle when the THI increases. Furthermore, the results show that the PTGS2 gene plays important roles in oxidative stress, oxidation-reduction processes, cellular oxidant detoxification, peroxidase activity, prostaglandin-endoperoxide synthase activity, and dioxygenase activity. It is possible that VEGF and PTGS2 expression in the VEGF signaling pathway promotes the production of antioxidants and enzymes.

Moreover, the results of this study show that the main signaling pathways are the AGE-RAGE pathway, the VEGF pathway, cytokine-cytokine receptor interactions, and TNF and HIF-1 pathways. These pathways mediate hypoxia responses and immunosuppression and are enriched for the VEGF and PTGS2 genes as well. Lee et al. [26] found that VEGF plays vital roles in hypoxia responses by controlling the expression of numerous hypoxia-responsive genes functioning in diverse processes of oxygen delivery. In addition, an investigation has shown that VEGF not only regulates oxygen supply and growth but also acts as a mediator of inflammatory cytokines [27].

Moreover, Ferrara et al. [28] have proven that in low-oxygen environments, VEGF can bind to the VEGF receptor on the endothelial cell membrane, causing receptor autophosphorylation. Therefore, it is possible that heat stress may cause immunosuppression, oxidative stress and endocrine disorder by significantly increasing VEGF and PTGS2 gene expression when the THI increases. In addition, heat may induce the VEGF signaling pathway to increase PTGS2 release. Camacho et al. [29] found that PTGS2 increases PGI_2_ release in human vascular cells. During exposure to inflammatory stimuli, blood vessels are also induced to contract by PGI_2_. Given that this study revealed that both the VEGF and PTGS2 genes are upregulated by THIs of 77, 82 and 88, it is possible that blood vessels in the lung exhibit changes in resistance during panting in dairy cattle.

## Conclusions

The present study shows that RR, pH, and pCO_2_ are significantly affected by the THI and that Jersey dairy cattle (*Bos taurus*) employ panting-type respiration to dissipate excess body heat when the THI is close to 90. A total of 212 DEGs were screened from different pairs of THI groups (THI77, THI82 and THI88). Furthermore, 16 heat-related DEGs were screened between 212 DEGs and 1370 heat-related genes. Importantly, four hub genes (ITBG3, PTGS2, VEGF, and MYL9) related to immunosuppression, oxidative stress, and endocrine disorder were identified from the 16 genes. It is possible that VEGF and PTGS2 upregulate the VEGF signaling pathway to improve oxygenation in dairy cattle under heat stress. These findings will help elucidate the underlying immune and oxygen regulation mechanisms of the VEGF signaling pathway in dairy cattle under heat stress.

## Methods

**Fig. 5:**
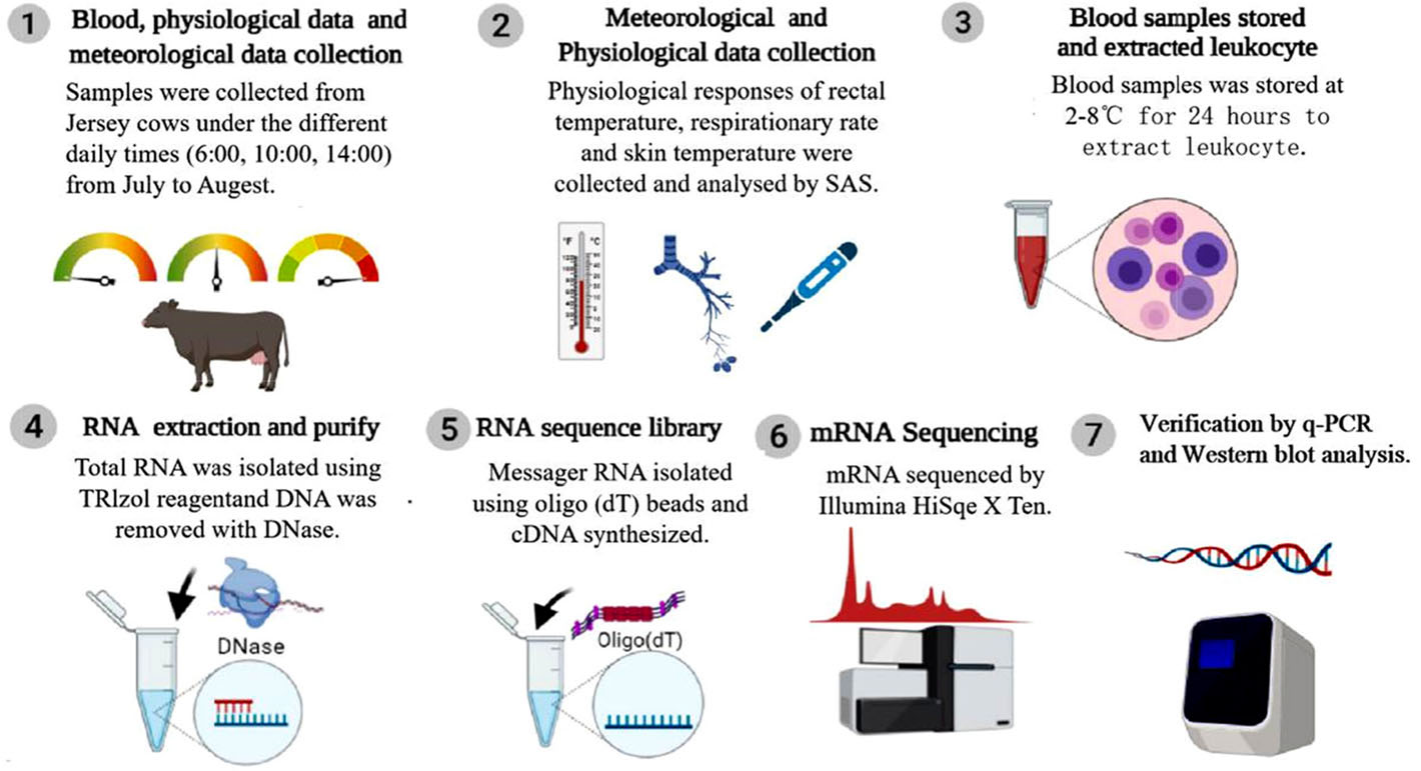
Diagram of the experimental design

This experiment was conducted with a random complete design (RCD) involving environmental temperature evaluation, identification of the physiological panting response, screening and analysis of heat-related differentially expressed genes, and gene verification (Fig. 5).

### Environment and experimental animals

Randomly selected healthy and dry Jersey dairy cattle (*n* = 5, 340 ± 10 kg, 4.5 years old) naturally exposed at hot environment in Chongqing (29.4316° N, 106.9123° E) were sampled continuously for 1 week from July to August of 2019 in this study.

### Physiological data for the physiological response test, blood sample collection for the gas exchange test and lymphocyte isolation

Physiological data including rectal temperature (RT), respiration rate (RR) and skin temperature (ST), as well as blood samples, were collected at 06:00, 10:00 and 14:00. Simultaneously, meteorological data were recorded using dry- and wet-bulb thermometers. The temperature and humidity index (THI) was calculated as described by Bohmanova et al. [30]. THI was calculated by the equation:

$$ \mathrm{THI}=\left(\mathrm{Tdb}+\right)\times 0.72+41 $$

Where Tdb is dry-bulb temperature and Twb is wet- bulb temperature.

RRs were determined by observing and counting the number of flank movements for 60 s. RT was measured using a digital clinical thermometer, whereas ST on skin with and without hair was measured with an infrared surface thermometer (Everest Interscience Inc., Fullerton, CA, USA). Blood pH and the partial pressure of carbon dioxide (pCO_2_) were measured with a Roche Omni C blood gas analyzer (MedWOW, UK). Whole-blood samples (10 ml) were obtained from the juguglar veins of Jersey dairy cattle and by coccygeal vein puncture with permission and were keptc with K2 EDTA anticoagulant to isolate lymphocytes. Briefly, blood samples were added to a tube (227,290, Greiner) and centrifuged at 1500 g at room temperature (18 °C) in a horizontal rotor (swing-out head) for a minimum of 20 min. After centrifugation, mononuclear cells and platelets were collected immediately. Mononuclear cells and cell pellets were resuspended by gentle vortexing with PBS and centrifuged at 1500 g for 10 min. The supernatant was aspirated as much as possible without disturbing the cell pellet, and then the lymphocytes were resuspended in PBS to isolate the cell pellet successfully for the subsequent procedure [31].

### RNA isolation, cDNA library construction and sequencing

Total RNA was isolated using TRIzol® Reagent according to the manufacturer’s protocol (Invitrogen, USA), and genomic DNA was removed using DNase I (Takara, Japan). RNA concentrations, purity and integrity were determined with a NanoDrop ND-2000 (NanoDrop Technologies, Inc., Wilmington, DE, USA) and with an RNA Nano 6000 Assay Kit on a Bioanalyzer 2100 system (Agilent Technologies, CA, USA). An RNA-seq transcriptome library was prepared using a TruSeq™ RNA sample preparation kit (Illumina, San Diego, CA, USA). Briefly, mRNA was isolated according to the polyA selection method with oligo (dT) beads and then fragmented with fragmentation buffer. Double-stranded cDNA was synthesized using a SuperScript Double-Stranded cDNA Synthesis Kit (Invitrogen, CA, USA) with random hexamer primers (Illumina, San Diego, CA, USA). Then, the synthesized cDNA was subjected to end repair, phosphorylation and ‘A’ base addition according to Illumina’s library construction protocol. The libraries were size-selected for cDNA target fragments of 200–300 bp on 2% Low Range Ultra Agarose and then subjected to PCR amplification using Phusion DNA polymerase (NEB, China) for 15 PCR cycles. After quantification with a TBS-380 instrument, the paired-end RNA-seq sequencing library was sequenced with the Illumina HiSeq X Ten (2 × 150 bp read length).

### Functional annotation and pathway analysis

Functional enrichment analyses including Gene Ontology (GO) and Kyoto Encyclopedia of Genes and Genomes (KEGG) analyses were performed to identify the significantly enriched GO terms and pathways for the DEGs. GO functional enrichment and KEGG pathway analysis were carried out with the GO R package GOATOOLS and KOBAS, respectively [32].

### Library data analysis

The raw data were first filtered. The obtained clean data were compared with the reference genome of the species. To identify DEGs between two different samples, the expression level of each transcript was calculated according to the fragments per kilobase of exon per million mapped reads (FPKM) method. RSEM was used to quantify gene abundances, and the expression of each gene was calculated. Then, differential expression analysis and KEGG and GO [33] enrichment analyses were performed with the Database for Annotation, Visualization and Integrated Discovery (DAVID) bioinformatics software (version 2.5.0) [34].

### Quantitative real-time polymerase chain reaction validation (Q-PCR)

To ensure the reliability of the mRNA-seq data, an RT Reagent Kit with gDNA Eraser (Takara, Chengdu, China) was used according to the manufacturer’s protocol. Q-PCR was performed on a StepOnePlus Real-Time PCR System (Life Technologies, Gaithersburg, MD, USA) according to standard methods using Fast Start Universal SYBR Green Master (ROX) (Roche, Mannheim, Germany). The relative expression of target genes was normalized to the expression of β-actin. The primers used for Q-PCR are given in Table 4. The relative gene expression values were calculated by the 2^-ΔΔCt^ method [35].

**Table 4:** Primers used for qRT-PCR

Gene	Accession number	Annealing temperature		Sequence (5′- > 3′)	Length	Tm
VEGFA	NM_001316955.1	65 °C	F	AAAGTCTGGAGTGTGTGCCC	20	60.18
			R	GCTGGCTTTGGTGAGGTTTG	20	59.97
TGFB2	XM_010813095.3		F	CAGAGTGCCTGAACAACGGA	20	60.25
			R	TCCCAGGTTCCTGTCTTTATGG	22	59.42
PRDM1	NM_001192936.1		F	CGGAGGCTTCCTTACCGAG	19	59.56
			R	CCTCTGGAATAGATCCGCCAAAA	23	60.43
SAMSN1	NM_001035404.1		F	TTCGACTGGGGTATGCGAAG	20	59.83
			R	TGTTCCGGTATGGGAGGGTA	20	59.66
BHLHE40	NM_001024929.1		F	AGGACAGCAAGGAGACCTACA	21	60.2
			R	ACTGCTTTTTCCAAGTGACCC	21	58.97
NR4A2	NM_001076208.1		F	TAAGTTTCCCTCGCCCCAAC	20	59.96
			R	CTAAAGCCCCCTGTCTCGTC	20	59.82
DUSP6	NM_001046195.1		F	TTAGGAGCCGCTGGACTTTT	20	59.31
			R	CGCTCGTTCTCGGTGTCAAT	20	60.73
EDN1	NM_181010.2		F	AAGAACTCAGGGACCAAGACTC	22	59.36
			R	CTGTTGCTGATGGCCTCCAA	20	60.61
MYL9	NM_001075234.1		F	GATGACTAGGCCATCCCAGC	20	59.96
			R	TCTCAGCCCGTTTCCTTCAC	20	59.97
PTGS2	NM_174445.2		F	ATCATTCACCAGGCAAAGGGC	21	61.24
			R	TTCCTTCTCTCCTGTAAGTTCCTCA	25	60.7
CXCL8	NM_173925.2		F	CTGGACAGCAGAGCTCACAA	21	59.97
			R	CTGGACAGCAGAGCTCACAA	20	60.04
IL1A	NM_174092.1		F	GCTCGGTTCAGCAAAGAAGT	20	58.77
			R	CGAAGTGGCTCATAGCTTGC	20	59.35
β-actin	AF191490.1		F	CAGAAGGACTCGTACGTGGG	20	59.83
			R	CCGTGCTCAATGGGGTACTT	20	60.04

### Heat-related DEG coexpression analysis and VEGF pathway visualization

Sixteen heat-related DEGs were identified by coexpression analysis with a bioinformatics software package in DAVID (version 2.0). The VEGF pathway was drawn with BioRender (https://biorender.com/).

### Immunoblotting

The samples were lysed and analyzed by immunoblotting according to a previously reported protocol [36]. Denatured protein samples (20 to 50 μg) in Laemmli buffer (Alfa Aesar, Heysham, UK) were separated on 4 to 20% sodium dodecyl sulfate-polyacrylamide gels and transferred by electroblotting onto nitrocellulose membranes (Bio-Rad, Watford, UK). The membranes were then incubated overnight at 4 °C with primary antibodies (diluted 1:1000 in PBS). The primary antibodies included anti-VEGF (catalog no. ab46154, Abcam), anti-PTGS2 (catalog no. ab169782, Abcam) and anti-β-Actin (clone AC-74, Sigma-Aldrich, Gillingham, UK). The secondary antibody was HRP-linked anti-rabbit IgG (polyclonal, Cell Signaling). The blots were incubated with SuperSignal West Dura substrate (Thermo), developed with Amersham Hyperfilm (GE Healthcare, Chalfont St. Giles, UK) or imaged with a Bio-Rad gel imager and analyzed using ImageJ or Image Lab (Bio-Rad). Semiquantitative densitometric analysis, the results of which are dependent on the detection method, exposure time and substrate reaction kinetics, does not imply linearity of the measurements. The resulting expression values supported the protein representation of the bands, increasing the reliability of the results.

### Statistical analysis

All data were statistically analyzed in SAS software [37] using one-way ANOVA. Analyses were conducted on both environmental and physiological data. In addition, the GO and KEGG pathway enrichment statistics were analyzed by Fisher’s exact test with the following cutoffs: a *p* < 0.05 was considered to indicate a significant difference, while a *p* < 0.01 was considered to indicate a highly significant difference. Furthermore, the Q-PCR validation and immunoblotting data were statistically analyzed using one-way ANOVA with Tukey post hoc testing. The experimental error is shown as the standard deviation, standard error of the mean or 95% confidence interval depending on the experiment. The significance levels are shown as follows: *, *p* ≤ 0.05; **, *p* ≤ 0.01; and ***, *p* ≤ 0.001.

## Supplementary Information

**Additional file 1.** The 16 genes enriched at GO and KEGG related to immunosuppression, oxidative stress, and endocrine disorder in lymphocytes under heat stress.

**Additional file 2.** Complemary material.

## Data Availability

The data of transcriptome analysis would show with excel involved total DEGs, up-regulation DEGs and down-regulation DEGs among THI77, THI82 and THI88; The 16 genes of heatmap, GO analysis, KEGG analysis, gene analysis, relative analysis.

## Abbreviations

VEGF: Vascular endothelial growth factor
DEGs: Differentially expressed genes
KEGG: Kyoto encyclopedia of genes and genomes
GO: Gene ontology
HIF-1: Hypoxia inducible factor-1
ITBG3: Integrin beta 3
OAS2: 2'-5'-oligoadenylate synthetase 2
MX2: MX dynamin like GTPase 2
IFIT5: Interferon induced protein with tetratricopeptide repeats 5
NR4A2: Nuclear receptor subfamily 4 group A member 2
CXCL8: C-X-C motif chemokine ligand 8
IL1A: Interleukin 1 alpha
DUP6: AF356445.1(Dual specificity phosphatase 6)
ICAM1: Intercellular adhesion molecule 1
END1: HNHc-like endonuclease
SAMSN1: SAM domain, SH3 domain and nuclear localization signals 1
BHLHE40: Basic helix-loop-helix family member e40
PTGS2: Prostaglandin-endoperoxide synthase 2
TGFB2: Transforming growth factor beta 2
DNAJB13: DnaJ heat shock protein family (Hsp40) member B13
TNF: Tumor necrosis factor
THI: Temperature humidity index
Hb: Hemoglobin
HbO_2_: Oxyhemoglobin
pO_2_: Partial pressure of oxygen
ANOVA: Analysis of variance
PPI: Protein–protein interaction
RT: Rectal temperature
RR: Respiratory rate
ST: Skin temperature
FRKM: Fragments per kilobase of exon per million mapped reads
MF: Molecular function
BP: Biological process
CC: Cellular component
CO_2_: Carbon dioxide
Bpm: Respiration rate unite:breath per minute
ml: Millilitre
°C: Degree Celsius
