# Supplementary material for: Involvement of the VEGF signaling pathway in immunosuppression and hypoxia stress: analysis of mRNA expression in lymphocytes mediating panting in Jersey cattle under heat stress
Source: BMC Vet Res. 2021 Jun 7;17:209. doi: 10.1186/s12917-021-02912-y (PMC8186226; doi:10.1186/s12917-021-02912-y)

**Additional file 1** It was involved experiments animal, quality control of analysis by PCA chart, volcano charts and a table of the distribution of the sequences.

1. Experiment animals.

| Cow ID | Year | Time(THI) | Breed | Weigh | BCS | Location |
| --- | --- | --- | --- | --- | --- | --- |
| 80/53 | 4 | 6:00(HIT77) | Jersey | 341 | 3 | Chongqing |
| 80/53 | 4 | 10:00(THI82) | Jersey | 341 | 3 | Chongqing |
| 80/53 | 4 | 14:00(THI88) | Jersey | 341 | 3 | Chongqing |
| 28/51 | 5 | 6:00(HIT77) | Jersey | 352 | 4 | Chongqing |
| 28/51 | 5 | 10:00(THI82) | Jersey | 352 | 4 | Chongqing |
| 28/51 | 5 | 14:00(THI88) | Jersey | 352 | 4 | Chongqing |
| 30/51 | 4 | 6:00(HIT77) | Jersey | 348 | 3.5 | Chongqing |
| 30/51 | 4 | 10:00(THI82) | Jersey | 348 | 3.5 | Chongqing |
| 30/51 | 4 | 14:00(THI88) | Jersey | 348 | 3.5 | Chongqing |
| 18/51 | 4 | 6:00(HIT77) | Jersey | 347 | 4 | Chongqing |
| 18/51 | 4 | 10:00(THI82) | Jersey | 347 | 4 | Chongqing |
| 18/51 | 4 | 14:00(THI88) | Jersey | 347 | 4 | Chongqing |
| 18/50 | 4 | 6:00(HIT77) | Jersey | 344 | 3 | Chongqing |
| 18/50 | 4 | 10:00(THI82) | Jersey | 344 | 3 | Chongqing |
| 18/50 | 4 | 14:00(THI88) | Jersey | 344 | 3 | Chongqing |

（2）data PCA analysis effected by different THI involved THI77, THI82 and THI88


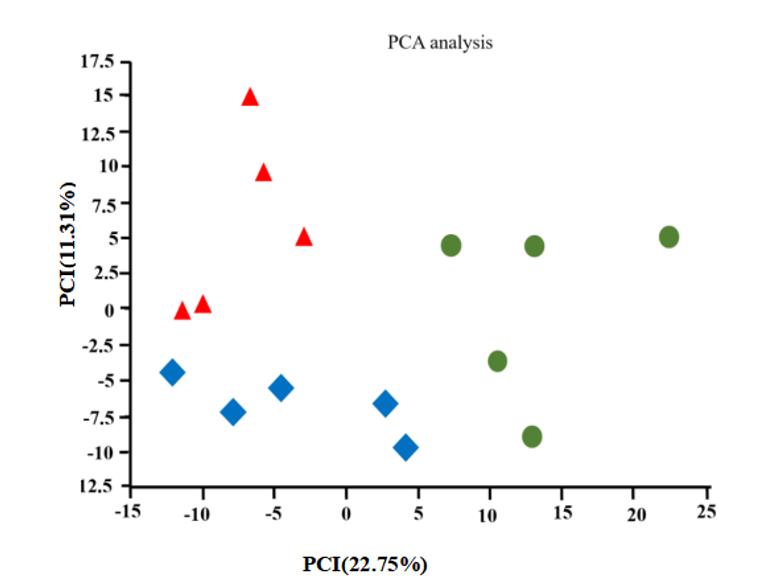


（3）Volcano charts to show different expression gene effected by different weather temperatures involved 25℃(THI77), 30℃(THI82) and 35℃(THI88)


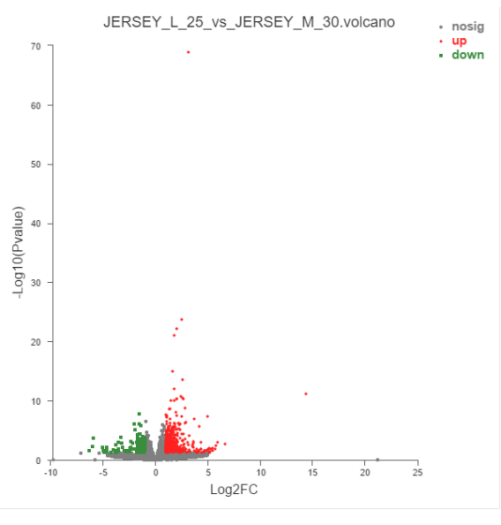

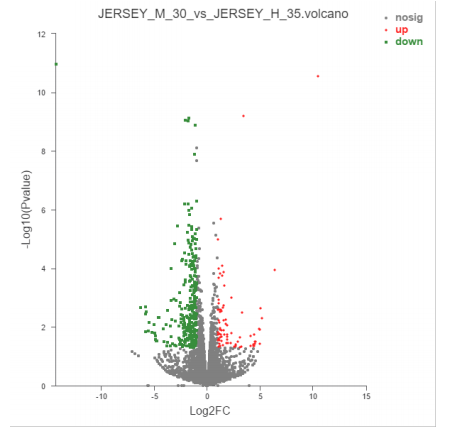


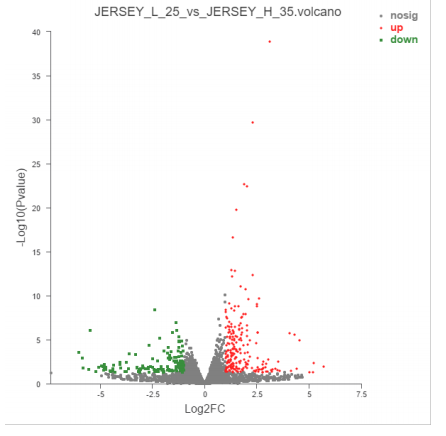


（4）Distribution of sequencing in different regions of the *Bos taurus* transcriptome.

| Sample | Introns (%) | 3'UTR (%) | CDS (%) | 5'UTR (%) | Intergenic (%) |
| --- | --- | --- | --- | --- | --- |
| THI=77_1 | 15.23 | 7.71 | 65.04 | 2.81 | 9.22 |
| THI=77_2 | 11.99 | 8.21 | 68.37 | 2.51 | 8.91 |
| THI=77_3 | 10.8 | 8.8 | 68.19 | 3.03 | 9.18 |
| THI=77_4 | 15.49 | 8.03 | 64.41 | 2.61 | 9.46 |
| THI=77_5 | 13.25 | 8.61 | 68.32 | 2.54 | 8.88 |
| THI=82_1 | 17.21 | 7.88 | 62.49 | 2.73 | 9.69 |
| THI=82_2 | 13.34 | 9.88 | 64.7 | 2.93 | 9.15 |
| THI=82_3 | 13.38 | 9.71 | 65.14 | 2.8 | 8.97 |
| THI=82_4 | 16.03 | 8.15 | 62.28 | 2.8 | 10.2 |
| THI=82_5 | 14.19 | 8.3 | 66.22 | 2.9 | 8.39 |
| THI=88_1 | 17.41 | 7.98 | 62.85 | 2.82 | 8.94 |
| THI=88_2 | 14.21 | 8.26 | 66.06 | 3.06 | 8.41 |
| THI=88_3 | 16.13 | 8.11 | 64.16 | 2.88 | 8.72 |
| THI=88_4 | 14.99 | 8.46 | 65.81 | 2.37 | 8.37 |
| THI=88_5 | 14.63 | 7.9 | 66.44 | 2.78 | 8.25 |

**Additional file 2** It shows the original analysis using normalized read counts effected by THI of 77(25℃), 82(30℃) and 88(35℃) within different expressed genes (DEGs) involved total DEGs, up-regulated DEGs and down-regulated DEGs, ,the 16 heat DEGs screened and showed its heatmap with data.


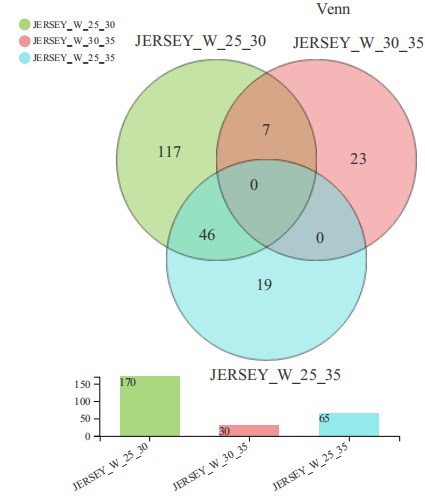


(A) Venn statistics shown total DEGs effected by each pair of THI group of pair of groups involved THI77 vs THI82, THI82 vs THI88 and THI77 vs THI88 ;


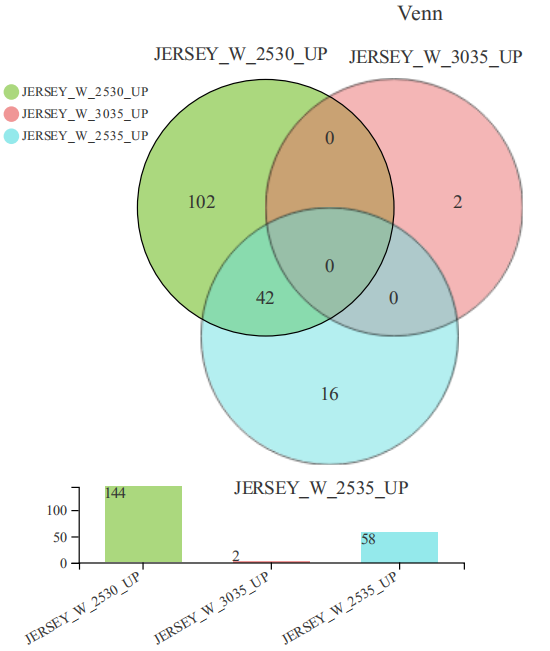


(B)Venn statistics shown up-regulated DEGs effected by each pair of THI involved THI77 vs THI82,THI82 vs THI88 and THI77 vs THI88;


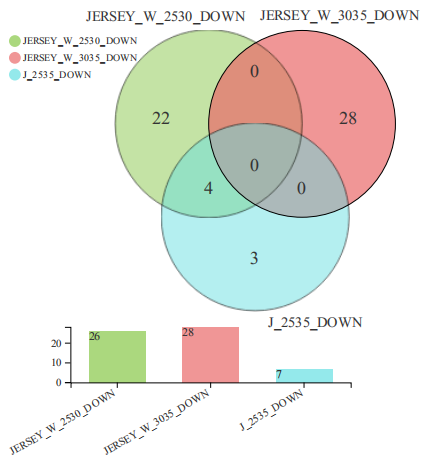


(C)Venn statistics shown down-regulated genes DEGs effected by each pair of THI group involved THI77 vs THI82, THI82 vs THI88 and THI77 vs THI88.


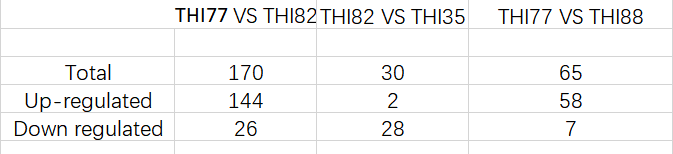


1. Statistic of the different expressed genes (DEGs) involved total DEGs, up-regulated DEGs and down-regulated DEGs.


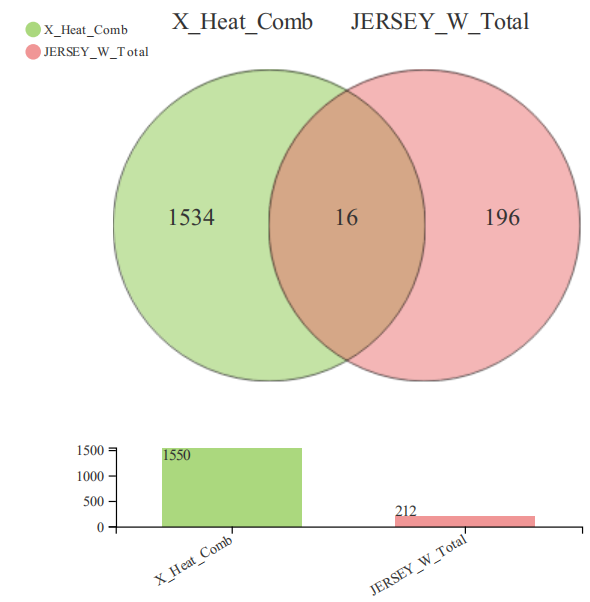


(E)The 16 heat DEGs was screened between heat genes and DEGs.


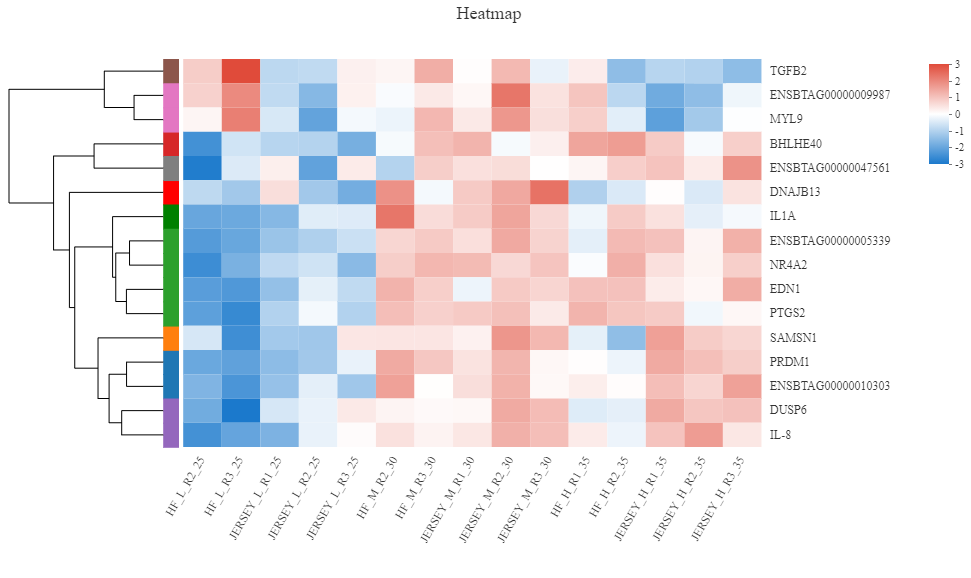

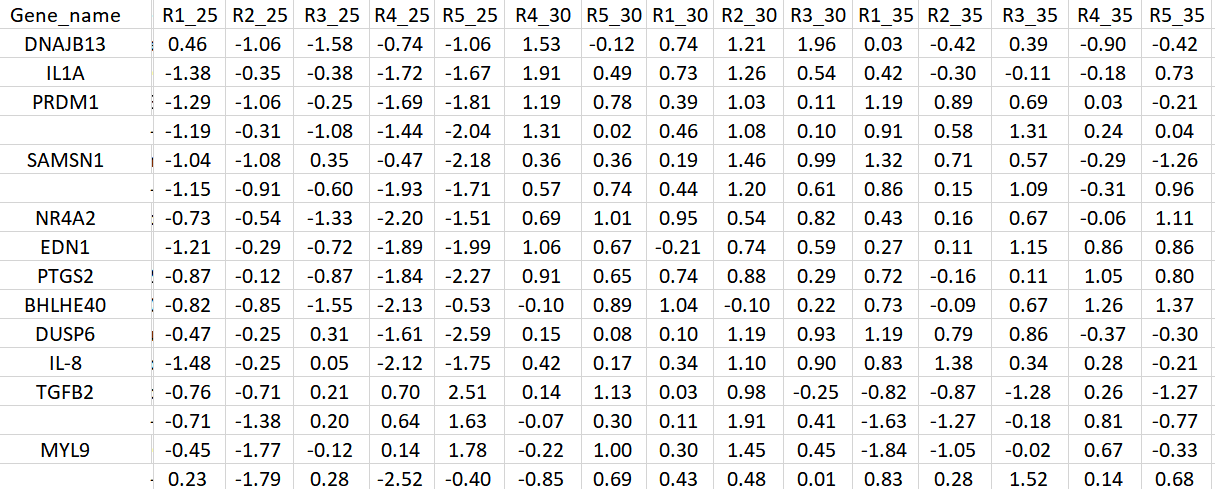


(F)There was shown Original heatmap and its data.

**Additional file 3**  The histogram of KEGG shows findings of genes in detail annotated at (a) signal transduction, (b) endocrine system, and (c)immune system.


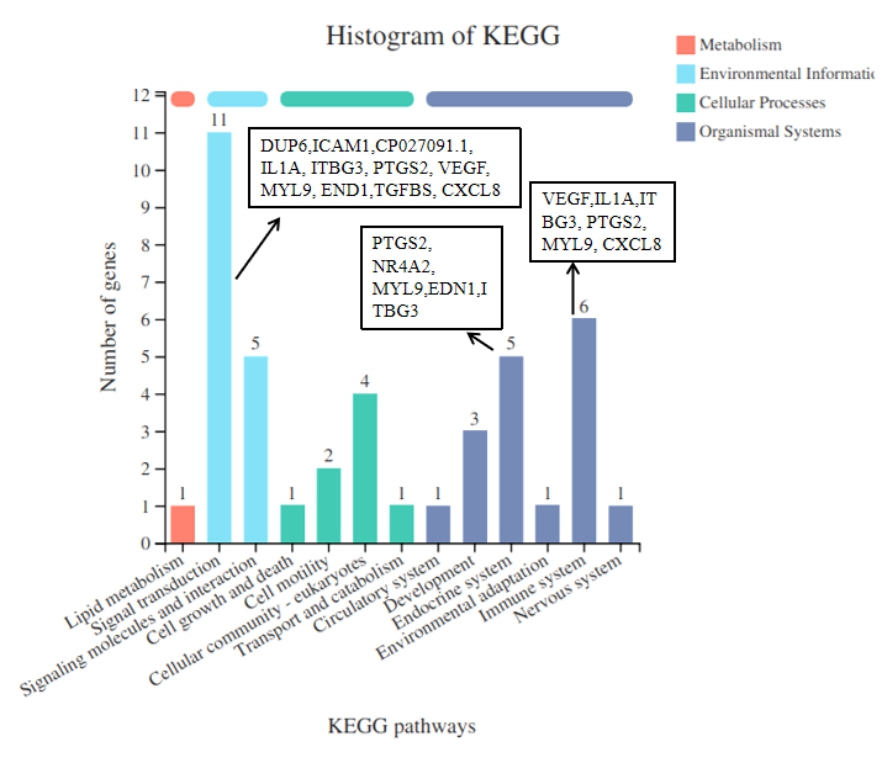


(a)


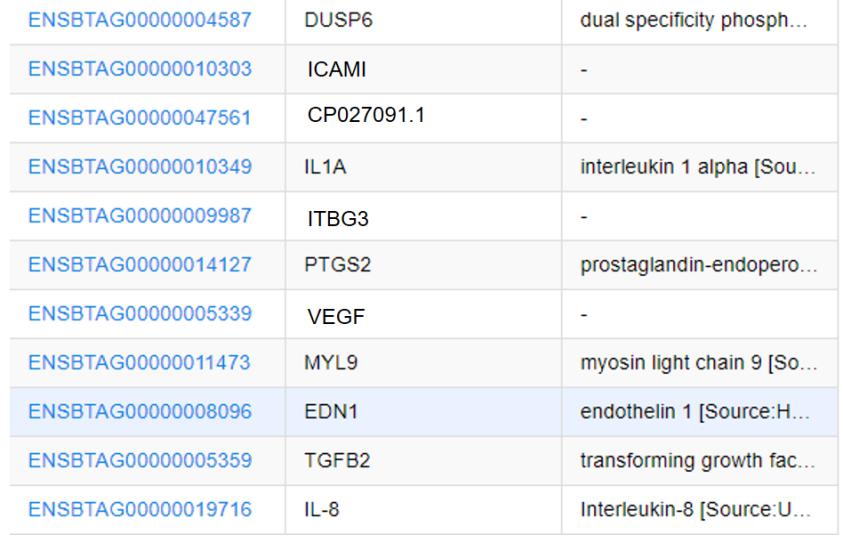


(b)


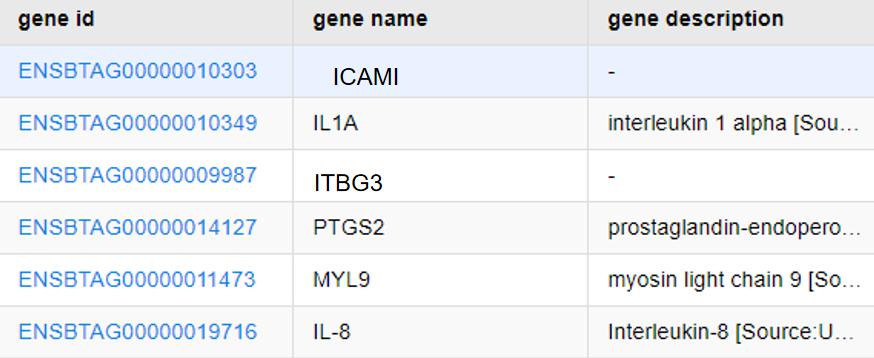


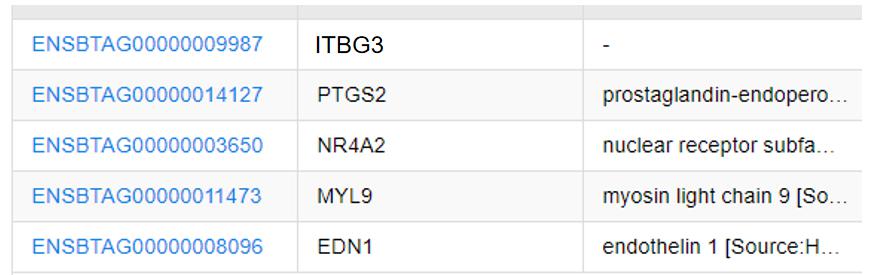
(c)

**Additional file 4** The Data supports heat DEGs expression between RNA-seq and Q-PCR.

| Gene | RNA-seq | Q-PCR |
| --- | --- | --- |
| VEGF | 3.116245633 | 5.171634024 |
| PRDM1 | 1.054091055 | 4.891134068 |
| SAMSN1 | 1.222218258 | 1.68605799 |
| BHLHE40 | 1.031179246 | 3.245455242 |
| NR4A2 | 1.523615429 | 1.959605925 |
| DUSP6 | 1.068648487 | 1.831203613 |
| EDN1 | 1.428660769 | 0.892913323 |
| MYL9 | 1.115241045 | 0.969697781 |
| PTGS2 | 1.581002521 | 0.730693962 |
| CXCL8 | 1.320893774 | 1.328946152 |
| TGFB2 | -1.117683905 | -0.459178681 |
| IL1A | -1.0851836 | -3.542810826 |

**Additional 5** Tables shows (1) raw data of gas exchanged at the fifth day ; (2) Raw data of physiological responses from day 1 to day 5.

| Cow ID | Year | Time(THI) | Day | pH | Pco_2_ |
| --- | --- | --- | --- | --- | --- |
| 80/53 | 4 | 6:00(HIT77) | 5 | 7.41 | 45 |
| 80/53 | 4 | 10:00(THI82) | 5 | 7.4 | 40.2 |
| 80/53 | 4 | 14:00(THI88) | 5 | 7.48 | 35 |
| 28/51 | 5 | 6:00(HIT77) | 5 | 7.44 | 37 |
| 28/51 | 5 | 10:00(THI82) | 5 | 7.45 | 39.6 |
| 28/51 | 5 | 14:00(THI88) | 5 | 7.44 | 31 |
| 30/51 | 4 | 6:00(HIT77) | 5 | 7.4 | 43 |
| 30/51 | 4 | 10:00(THI82) | 5 | 7.45 | 39.8 |
| 30/51 | 4 | 14:00(THI88) | 5 | 7.46 | 30 |
| 18/51 | 4 | 6:00(HIT77) | 5 | 7.42 | 38 |
| 18/51 | 4 | 10:00(THI82) | 5 | 7.5 | 39.9 |
| 18/51 | 4 | 14:00(THI88) | 5 | 7.49 | 33 |
| 18/50 | 4 | 6:00(HIT77) | 5 | 7.38 | 41 |
| 18/50 | 4 | 10:00(THI82) | 5 | 7.45 | 40.1 |
| 18/50 | 4 | 14:00(THI88) | 5 | 7.43 | 36 |

| Cow ID | day | Time(THI) | Ambient temp (°C) | Humidity | | Skin temperature | Retcal temp （°C) | Respiration rate |
| --- | --- | --- | --- | --- | --- | --- | --- | --- |
|  |  |  |  | Dry bulb | Wet bulb |  |  |  |
| 80/53 | 1 | 6:00(HIT77) | 26.4 | 26.1 | 25 | 36 | 38.5 | 51 |
| 80/53 | 1 | 10:00(THI82) | 29.3 | 30 | 27 | 38 | 38.8 | 77 |
| 80/53 | 1 | 14:00(THI88) | 36 | 35 | 35.1 | 39.6 | 39.5 | 121 |
| 28/51 | 1 | 6:00(HIT77) | 26.4 | 26.1 | 25 | 37.5 | 38.9 | 53 |
| 28/51 | 1 | 10:00(THI82) | 29.3 | 30 | 27 | 38.4 | 38.4 | 74 |
| 28/51 | 1 | 14:00(THI88) | 36 | 35 | 35.1 | 39.5 | 39.6 | 123 |
| 30/51 | 1 | 6:00(HIT77) | 26.4 | 26.1 | 25 | 35.5 | 38.1 | 49 |
| 30/51 | 1 | 10:00(THI82) | 29.3 | 30 | 27 | 38.6 | 38.8 | 78 |
| 30/51 | 1 | 14:00(THI88) | 36 | 35 | 35.1 | 39.3 | 39.8 | 119 |
| 18/51 | 1 | 6:00(HIT77) | 26.4 | 26.1 | 25 | 36.2 | 38.7 | 54 |
| 18/51 | 1 | 10:00(THI82) | 29.3 | 30 | 27 | 38 | 38.6 | 76 |
| 18/51 | 1 | 14:00(THI88) | 36 | 35 | 35.1 | 39.4 | 39.3 | 124 |
| 46/50 | 1 | 6:00(HIT77) | 26.4 | 26.1 | 25 | 35.8 | 38.3 | 48 |
| 46/50 | 1 | 10:00(THI82) | 29.3 | 30 | 27 | 38.2 | 38.7 | 75 |
| 46/50 | 1 | 14:00(THI88) | 36 | 35 | 35.1 | 39.6 | 39.6 | 164 |
| 80/53 | 2 | 6:00(HIT77) | 26.9 | 26.4 | 26 | 36 | 38.5 | 51 |
| 80/53 | 2 | 10:00(THI82) | 30.2 | 29 | 26.5 | 38 | 38.8 | 77 |
| 80/53 | 2 | 14:00(THI88) | 35.5 | 36 | 35.4 | 39.6 | 39.5 | 121 |
| 28/51 | 2 | 6:00(HIT77) | 26.9 | 26.4 | 26 | 37.5 | 38.9 | 53 |
| 28/51 | 2 | 10:00(THI82) | 30.2 | 29 | 26.5 | 38.4 | 38.4 | 74 |
| 28/51 | 2 | 14:00(THI88) | 35.5 | 36 | 35.4 | 39.5 | 39.6 | 123 |
| 30/51 | 2 | 6:00(HIT77) | 26.9 | 26.4 | 26 | 35.5 | 38.1 | 49 |
| 30/51 | 2 | 10:00(THI82) | 30.2 | 29 | 26.5 | 38.6 | 38.8 | 78 |
| 30/51 | 2 | 14:00(THI88) | 35.5 | 36 | 35.4 | 39.3 | 39.8 | 119 |
| 18/51 | 2 | 6:00(HIT77) | 26.9 | 26.4 | 26 | 36.2 | 38.8 | 55 |
| 18/51 | 2 | 10:00(THI82) | 30.2 | 29 | 26.5 | 38 | 38.6 | 76 |
| 18/51 | 2 | 14:00(THI88) | 35.5 | 36 | 35.4 | 39.4 | 39.3 | 132 |
| 46/50 | 2 | 6:00(HIT77) | 26.9 | 26.4 | 26 | 35.8 | 38.3 | 48 |
| 46/50 | 2 | 10:00(THI82) | 30.2 | 29 | 26.5 | 38.2 | 38.6 | 75 |
| 46/50 | 2 | 14:00(THI88) | 35.5 | 36 | 35.4 | 39.6 | 39.6 | 164 |
| 80/53 | 3 | 6:00(HIT77) | 26.6 | 25.7 | 25.2 | 36 | 38.5 | 51 |
| 80/53 | 3 | 10:00(THI82) | 28 | 29.5 | 26.5 | 38 | 38.8 | 77 |
| 80/53 | 3 | 14:00(THI88) | 36.5 | 35.5 | 35.3 | 39.6 | 39.5 | 121 |
| 28/51 | 3 | 6:00(HIT77) | 26.6 | 25.7 | 25.2 | 37.5 | 38.9 | 53 |
| 28/51 | 3 | 10:00(THI82) | 28 | 29.5 | 26.5 | 38.4 | 38.4 | 73 |
| 28/51 | 3 | 14:00(THI88) | 36.5 | 35.5 | 35.3 | 39.5 | 39.6 | 123 |
| 30/51 | 3 | 6:00(HIT77) | 26.6 | 25.7 | 25.2 | 35.5 | 38.1 | 49 |
| 30/51 | 3 | 10:00(THI82) | 28 | 29.5 | 26.5 | 38.6 | 38.8 | 78 |
| 30/51 | 3 | 14:00(THI88) | 36.5 | 35.5 | 35.3 | 39.3 | 39.8 | 119 |
| 18/51 | 3 | 6:00(HIT77) | 26.6 | 25.7 | 25.2 | 36.2 | 38.5 | 54 |
| 18/51 | 3 | 10:00(THI82) | 28 | 29.5 | 26.5 | 38 | 38.6 | 76 |
| 18/51 | 3 | 14:00(THI88) | 36.5 | 35.5 | 35.3 | 39.4 | 39.3 | 131 |
| 46/50 | 3 | 6:00(HIT77) | 26.6 | 25.7 | 25.2 | 35.8 | 38.3 | 48 |
| 46/50 | 3 | 10:00(THI82) | 28 | 29.5 | 26.5 | 38.2 | 38.9 | 75 |
| 46/50 | 3 | 14:00(THI88) | 36.5 | 35.5 | 35.3 | 39.6 | 39.6 | 164 |
| 80/53 | 4 | 6:00(HIT77) | 26.8 | 26 | 25.4 | 36 | 38.5 | 51 |
| 80/53 | 4 | 10:00(THI82) | 32.2 | 30 | 26.5 | 38 | 38.8 | 77 |
| 80/53 | 4 | 14:00(THI88) | 36.2 | 36 | 35.2 | 39.6 | 39.5 | 121 |
| 28/51 | 4 | 6:00(HIT77) | 26.8 | 26 | 25.4 | 37.5 | 38.9 | 53 |
| 28/51 | 4 | 10:00(THI82) | 32.2 | 30 | 26.5 | 38.4 | 38.4 | 76 |
| 28/51 | 4 | 14:00(THI88) | 36.2 | 36 | 35.2 | 39.5 | 39.6 | 123 |
| 30/51 | 4 | 6:00(HIT77) | 26.8 | 26 | 25.4 | 35.5 | 38.1 | 49 |
| 30/51 | 4 | 10:00(THI82) | 32.2 | 30 | 26.5 | 38.6 | 38.8 | 78 |
| 30/51 | 4 | 14:00(THI88) | 36.2 | 36 | 35.2 | 39.3 | 39.8 | 119 |
| 18/51 | 4 | 6:00(HIT77) | 26.8 | 26 | 25.4 | 36.2 | 38.5 | 54 |
| 18/51 | 4 | 10:00(THI82) | 32.2 | 30 | 26.5 | 38 | 38.6 | 76 |
| 18/51 | 4 | 14:00(THI88) | 36.2 | 36 | 35.2 | 39.4 | 39.3 | 126 |
| 46/50 | 4 | 6:00(HIT77) | 26.8 | 26 | 25.4 | 35.8 | 38.3 | 48 |
| 46/50 | 4 | 10:00(THI82) | 32.2 | 30 | 26.5 | 38.2 | 38.7 | 71 |
| 46/50 | 4 | 14:00(THI88) | 36.2 | 36 | 35.2 | 39.6 | 39.6 | 164 |
| 80/53 | 5 | 6:00(HIT77) | 26.7 | 26 | 24.4 | 36 | 38.5 | 51 |
| 80/53 | 5 | 10:00(THI82) | 30.2 | 29 | 26 | 38 | 38.8 | 77 |
| 80/53 | 5 | 14:00(THI88) | 35.8 | 35.5 | 35.4 | 39.6 | 39.5 | 121 |
| 28/51 | 5 | 6:00(HIT77) | 26.7 | 26 | 24.4 | 37.5 | 38.9 | 53 |
| 28/51 | 5 | 10:00(THI82) | 30.2 | 29 | 26 | 38.4 | 38.4 | 74 |
| 28/51 | 5 | 14:00(THI88) | 35.8 | 35.5 | 35.4 | 39.5 | 39.6 | 131 |
| 30/51 | 5 | 6:00(HIT77) | 26.7 | 26 | 24.4 | 35.5 | 38.1 | 49 |
| 30/51 | 5 | 10:00(THI82) | 30.2 | 29 | 26 | 38.6 | 38.8 | 78 |
| 30/51 | 5 | 14:00(THI88) | 35.8 | 35.5 | 35.4 | 39.3 | 39.8 | 119 |
| 18/51 | 5 | 6:00(HIT77) | 26.7 | 26 | 24.4 | 36.2 | 38.7 | 54 |
| 18/51 | 5 | 10:00(THI82) | 30.2 | 29 | 26 | 38 | 38.6 | 76 |
| 18/51 | 5 | 14:00(THI88) | 35.8 | 35.5 | 35.4 | 39.4 | 39.3 | 125 |
| 46/50 | 5 | 6:00(HIT77) | 26.7 | 26 | 24.4 | 35.8 | 38.3 | 48 |
| 46/50 | 5 | 10:00(THI82) | 30.2 | 29 | 26 | 38.2 | 38.4 | 75 |
| 46/50 | 5 | 14:00(THI88) | 35.8 | 35.5 | 35.4 | 39.6 | 39.6 | 164 |

| gene name  **Additional 6** The 16 genes enriched at GO and KEGG related to immunosuppression, oxidative stress, and endocrine disorder in lymphocytes under heat stress. | GO_id | GO_term | KO_id | KO_name | Pathway_id | Pathway_definition |
| --- | --- | --- | --- | --- | --- | --- |
| TGFB2 | GO:0001666  GO:0010629 | BP: response to hypoxia  BP: negative regulation of gene expression | K13376 | TGFB2 | map04060  Map04350 | Cytokine-cytokine receptor interaction  TGF-beta signaling pathway |
| ITBG3 | GO:0004872  GO:0019899 | MF::receptor activity  MF:enzyme biinding | K06493 | ITGB3,CD61 | map04919  map04151 | Thyroid hormone signaling pathway  PI3K-Akt signaling pathway |
| DNAJB13 | GO:0006457 GO:0051082 | BP:protein folding MF:unfolded protein binding | K09519 | DNAJB13 | ------ | ------ |
| IL1A | GO:0006955 GO:0019221 GO:0034605 GO:0035234 GO:0050715 GO:0005125 GO:0005149 | **BP:immune response** **BP:cytokine-mediated signaling pathway** **BP:cellular response to heat** BP:ectopic germ cell programmed cell death **BP:positive regulation of cytokine secretion** **MF:cytokine activity** MF:interleukin-1 receptor binding | K04383 | IL1A | map04933 map04010 map04060 | AGE-RAGE signaling pathway in diabetic complications; **MAPK signaling pathway; Cytokine-cytokine receptor interaction.** |
| PRDM1 | GO:0000122 GO:0010628 | BP:Negative regulation of transcription from RNA polymerase II promoter BP:positive regulation of gene expression | ------ | ------ | ------ | ------ |
| SAMSN1 | GO:0002820 GO:0050732 GO:0050869 GO:0005634 GO:0005829 GO:0001784 | **BP:Negative regulation of adaptive immune response** **BP:Negative regulation of peptidyl-tyrosine phosphorylation** BP:Negative regulation of B cell activation CC:Nucleus CC:Cytosol MP:Phosphotyrosine binding | ------ | ------ | ------ | ------ |
| VEGFA | GO:0007595 GO:0032147 GO:0038033  GO:0038091  GO:0038190 GO:0043117 GO:0050927 GO:0050930 GO:0060749 | BP:Lactation BP:Activation of protein kinase activity **BP:Positive regulation of endothelial cell chemotaxis by VEGF-activated vascular endothelial growth factor receptor signaling BP:Positive regulation of cell proliferation by VEGF-activated platelet derived growth factor receptor signaling pathway** **BP:VEGF-activated neurophil signaling pathway** BP:Positive regulation of vascular permeability BP:Positive regulation of positive chemotaxis BP:Induction of positive chemotaxis BP:Mammary gland alveolus development **BP:Cellular response to hypoxia MF:Cytokine activity** | K05448 | VEGFA | map04015 map04014 map04933 map04370 map04060 map04151 map04066 | Rap1 signaling pathway Ras signaling pathway AGE-RAGE signaling pathway in diabetic complications **VEGF signaling pathway** **Cytokine-cytokine receptor interaction** PI3K-Akt signaling pathway **HIF-1 signaling pathway** |
| EDN1 | GO:0001569 GO:0001666 GO:0007585 GO:0007589 GO:0014065 GO:0019229 GO:0030072 GO:0035815 GO:0005125 GO:0005179 | BP:Patterning of blood vessels **BP:Response to hypoxia** BP:Regulation of pH BP:Body fluid secretion BP:Phosphatidylinositol 3-kinase signaling BP:Regulation of vasoconstriction BP:Peptide hormone secretion BP:Positive regulation of renal sodium excretion **MF:Cytokine activity** MF:Hormone activity | K16366 | EDN1 | map04270 map04668 map04066 map04933 map05410 | Vascular smooth muscle contraction **TNF signaling pathway HIF-1 signaling pathway** AGE-RAGE signaling pathway in diabetic complications Hypertrophic cardiomyopathy (HCM) |
| ICAM1 | GO:0016337 GO:0005887 GO:0016021 GO:0005178 | BP:Single organismal cell-cell adhesion CC:Integral component of plasma membrane CC:Integral component of membrane MF:Integrin binding | K06490 | ICAM1 CD54 | map04514 map04650 map04668 map04670 map04064 map04933 | Cell adhesion molecules (CAMs) **Natural killer cell mediated cytotoxicity TNF signaling pathway** Leukocyte transendothelial migration NF-kappa B signaling pathway AGE-RAGE signaling pathway in diabetic complications |
| BHLHE40 | GO:0000122 GO:0032922 | BP:circadian regulation of gene expression BP:negative regulation of transcription from RNA polymerase II promoter | K03729 | BHLHB2, DEC1 | map04710 | Circadian rhythm |
| NR4A2 | GO:0001666 GO:0006367 GO:0034599 GO:0043576 | **BP:Response to hypoxia** BP:Transcription initiation from RNA polymerase II promoter **BP:Cellular response to oxidative stress BP:Regulation of respiratory gaseous exchange** | K08558 | NR4A2, NURR1 | map04925 | Aldosterone synthesis and secretion |
| DUSP6 | GO:0000188 GO:0043065 GO:0070373 GO:0004725 | **BP:Inactivation of MAPK activity** BP:Positive regulation of apoptotic process BP:Negative regulation of ERK1 and ERK2 cascade **MF:Protein tyrosine phosphatase activity** | K04459 | DUSP, MKP | map04010 | **MAPK signaling pathway** |
| MYL9 | GO:0070527 GO:0001725 GO:0016460 GO:0030018 GO:0005509 GO:0032036 | BP：Platelet aggregation CC：Stress fiber CC：Myosin II complex CC：Z disc MF：Calcium ion binding MF：Myosin heavy chain binding | K12755 | MYL9 | map04921 map04510 map04810 map04270 map04024 map04022 map04670 | Oxytocin signaling pathway Focal adhesion Regulation of actin cytoskeleton Vascular smooth muscle contraction cAMP signaling pathway cGMP-PKG signaling pathway Leukocyte transendothelial migration |
| PTGS2 | GO:0001934 GO:0006954 GO:0006979 GO:0055114 GO:0071347 GO:0098869 GO:0004601 GO:0004666 | **BP:Inflammatory response BP:Response to oxidative stress BP:Oxidation-reduction process BP:Cellular response to interleukin-1 BP:Cellular oxidant detoxification MF:Peroxidase activity MF:Prostaglandin-endoperoxide synthase activity MF:Dioxygenase activity** | K11987 | PTGS2, COX2 | map04657 map04064 map04668 map04921 map04370 map04726 map04913 | IL-17 signaling pathway NF-kappa B signaling pathway **TNF signaling pathway** Oxytocin signaling pathway **VEGF signaling pathway** Serotonergic synapse Ovarian steroidogenesis |
| CXCL8 | GO:0006954 GO:0006955 GO:0030593 GO:0050930 GO:0070098 GO:0071347 GO:0090023 GO:0005153 GO:0008009 | BP:Inflammatory response **BP:Immune response** BP:Neutrophil chemotaxis BP:Induction of positive chemotaxis BP:Chemokine-mediated signaling pathway BP:Cellular response to interleukin-1 BP:Positive regulation of neutrophil chemotaxis MF:Interleukin-8 receptor binding **MF:Chemokine activity** | K10030 | IL8, CXCL8 | map04657 map04622 map04620 map04621 map04060 map04062 map04064 | IL-17 signaling pathway RIG-I-like receptor signaling pathway Toll-like receptor signaling pathway NOD-like receptor signaling pathway **Cytokine-cytokine receptor interaction** Chemokine signaling pathway NF-kappa B signaling pathway |
| CP027091.1 | GO:0038033  GO:0038091  GO:0045944 GO:0050731 GO:0071456 | BP：Positive regulation of endothelial cell chemotaxis by VEGF-activated vascular endothelial growth factor receptor signaling pathway BP：Positive regulation of cell proliferation by VEGF-activated platelet derived growth factor receptor signaling pathway BP：Positive regulation of transcription from RNA polymerase II promoter BP：Positive regulation of peptidyl-tyrosine phosphorylation BP：Positive regulation of positive chemotaxis **BP：Cellular response to hypoxia**  **MF: Cytokine activity** | K05448 | VEGFA | map04933 map04370 map04060 map01521 map04151 map04066 | AGE-RAGE signaling pathway in diabetic complications **VEGF signaling pathway** **Cytokine-cytokine receptor interaction** **EGFR tyrosine kinase inhibitor resistance** PI3K-Akt signaling pathway **HIF-1 signaling pathway** |

**Additional 7** It shows （A）Co-expression network diagram of the 16 heat DEG and (B) the diagram of VEGF signaling pathway.

(A)


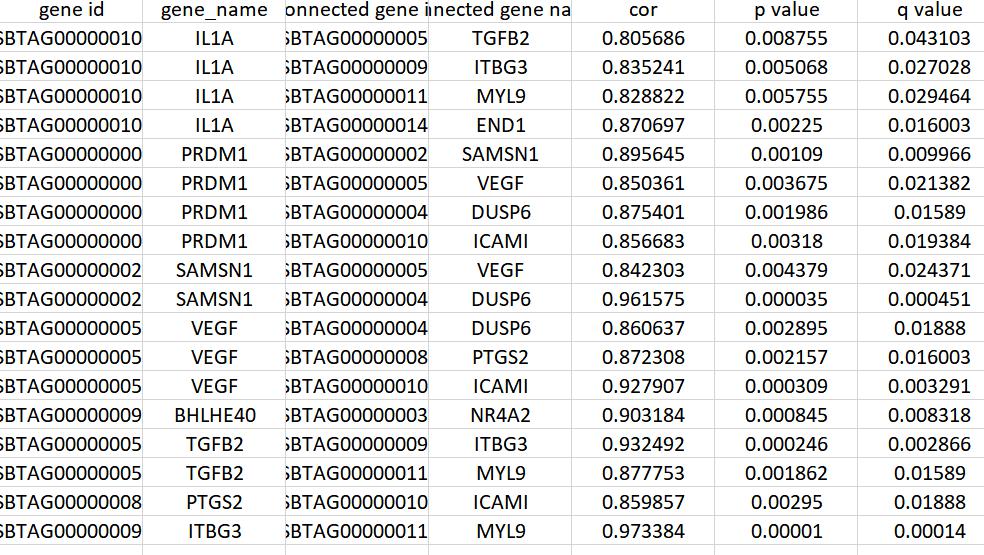


(B)


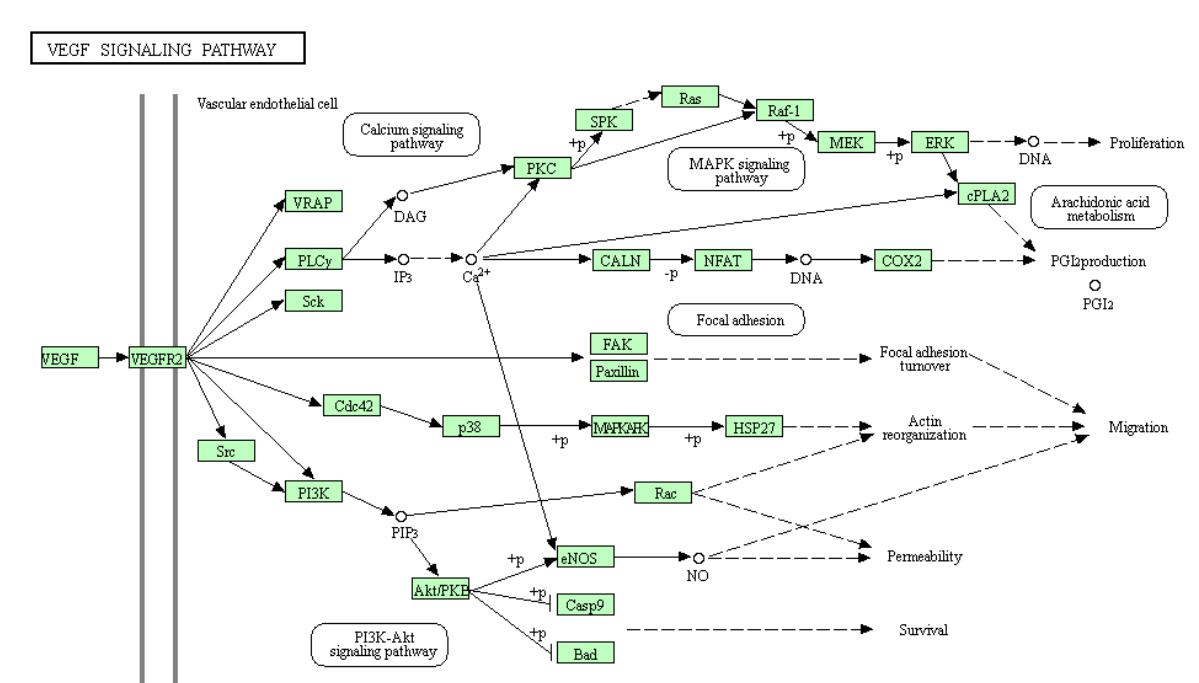

Supplement: Supplementary file 1 — Additional file 1. The 16 genes enriched at GO and KEGG related to immunosuppression, oxidative stress, and endocrine disorder in lymphocytes under heat stress. [file 12917_2021_2912_MOESM1_ESM.docx]
